# Photoinduced isomerization sampling of retinal in bacteriorhodopsin

**DOI:** 10.1093/pnasnexus/pgac103

**Published:** 2022-07-01

**Authors:** Zhong Ren

**Affiliations:** Department of Chemistry, University of Illinois at Chicago, Chicago, IL 60607, USA; Renz Research, Inc., Westmont, IL 60559, USA

**Keywords:** charge separation, proton pump, serial crystallography, singular value decomposition, X-ray free electron laser

## Abstract

Photoisomerization of retinoids inside a confined protein pocket represents a critical chemical event in many important biological processes from animal vision, nonvisual light effects, to bacterial light sensing and harvesting. Light-driven proton pumping in bacteriorhodopsin entails exquisite electronic and conformational reconfigurations during its photocycle. However, it has been a major challenge to delineate transient molecular events preceding and following the photoisomerization of the retinal from noisy electron density maps when varying populations of intermediates coexist and evolve as a function of time. Here, I report several distinct early photoproducts deconvoluted from the recently observed mixtures in time-resolved serial crystallography. This deconvolution substantially improves the quality of the electron density maps, hence demonstrates that the all-*trans* retinal undergoes extensive isomerization sampling before it proceeds to the productive 13-*cis* configuration. Upon light absorption, the chromophore attempts to perform *trans*-to-*cis* isomerization at every double bond together with the stalled *anti*-to-*syn* rotations at multiple single bonds along its polyene chain. Such isomerization sampling pushes all seven transmembrane helices to bend outward, resulting in a transient expansion of the retinal binding pocket, and later, a contraction due to recoiling. These ultrafast responses observed at the atomic resolution support that the productive photoreaction in bacteriorhodopsin is initiated by light-induced charge separation in the prosthetic chromophore yet governed by stereoselectivity of its protein pocket. The method of a numerical resolution of concurrent events from mixed observations is also generally applicable.

Significance Statement Photoisomerization of retinal is a critical rearrangement reaction in many important biological processes from animal vision, nonvisual light effects, to bacterial light sensing and harvesting. It has been a major challenge to visualize rapid molecular events preceding and following photoisomerization so that many protein functions depending on such reaction remain vaguely understood. Here, I report a direct observation of the stereoselectivity of bacteriorhodopsin, hence delineate the structural mechanism of isomerization. Upon a light-induced charge separation, the retinal in a flat conformation attempts to perform double bond isomerization and single bond rotation everywhere along its polyene chain before it proceeds to the specific, productive configuration. This observation improves our understanding on how a nonspecific attraction force could drive a specific isomerization.

## Introduction

Bacteriorhodopsin (bR) pumps protons outward from the cytoplasm against the concentration gradient via photoisomerization of its retinal chromophore. The trimeric bR on the native purple membrane shares the seven transmembrane helical fold and the same prosthetic group (Supplementary Fig. S1) with large families of microbial and animal rhodopsins (1, 2). An all-*trans* retinal in the resting state is covalently linked to Lys216 of helix G through a protonated Schiff base (SB; Supplementary Fig. S1e), of which the double bond C_15_=N_ζ_ is also in *trans* (traditionally also noted as *anti* (3)). Upon absorption of a visible photon, the all-*trans* retinal in bR isomerizes efficiently and selectively to adopt the 13-*cis* configuration (4, 5). In contrast, an all-*trans* free retinal in organic solvents could isomerize about various double bonds with a preference to form 11-*cis*, but much slower (6, 7) and with poor quantum yields (8, 9).

A broad consensus is that the isomerization event takes place around 450 to 500 fs during the transition from a blue-shifted species I to form a red-shifted intermediate J (10, 11). This ultrafast event is explained in excited state dynamics as a barrierless reaction, or at least with a very small activation barrier, from the Frank–Condon point to the conical intersection between the potential energy surfaces of the ground and excited states (12, 13). Various molecular events prior to the isomerization have also been detected. Vibrational spectroscopy showed a variety of possible motions, such as torsions about C_13_=C_14_ and C_15_=N_ζ_, H-out-of-plane wagging at C_14_, and even protein responses (14, 15). Nevertheless, the species I or a collection of species detected before 30 fs remain in a good *trans* configuration about C_13_=C_14_ instead of a near 90° configuration (16). Recently, deep-UV stimulated Raman spectroscopy revealed strong signals of Trp and Tyr motions in the protein throughout the I and J intermediates (17). Despite extensive studies, fundamental questions on the photoisomerization of retinal remain unanswered at the atomic resolution. It is assumed that the specific isomerization at C_13_=C_14_ in bR occurs faster with a high quantum yield compared to the slower, nonspecific isomerization at multiple sites for free retinal in solution and gas phase (6–9, 18) because the interactions between the retinal chromophore and its protein environment somehow catalyze the photoreaction by tuning the potential energy surfaces of the ground and/or excited states. What is the structural basis of such tuning or catalysis? What is the quantum mechanical force that causes the all-*trans* retinal to isomerize specifically to 13-*cis* in bR? Why not isomerize elsewhere in bR? How is the quantum yield of this specific isomerization enhanced by the protein compared to those of free retinal in solution? Does any isomerization sampling occur, that is, is isomerization even attempted at other double bonds? This work addresses these questions by solving a series of structures of the early intermediates based on the electron density maps unscrambled from the published serial crystallography datasets using singular value decomposition (SVD). These structures of “pure” photoproducts at atomic resolution reveal widespread conformational changes in all seven helices prior to the all-*trans* to 13-*cis* isomerization and after its completion, suggesting that isomerization sampling takes place in bR, where rapid photoisomerizations and single bond rotations are attempted everywhere along the polyene chain of the retinal before the only successful one flips the SB at ∼500 fs. The implication of these findings to the proton pumping and directional conductance is presented in a companion paper (19).

Several international consortia carried out large operations of serial crystallography at X-ray free electron lasers (XFELs). It is now possible to capture transient structural species at room temperature in the bR photocycle as short-lived as fs (20). Compared to cryo-trapping, authentic structural signals from these XFEL data are expected to be greater in both amplitude and scope. However, the signals reported so far do not appear to surpass those obtained by cryo-trapping methods, suggesting much needed improvements in experimental protocols and data analysis methods. Two major sources of data are used in this study (Supplementary Table S1). Nogly et al. captured retinal isomerization to 13-*cis* by the time of 10 ps and attributed its specificity to the H-bond breaking between the SB and a water (21). Kovacs et al. deposited datasets at many short time delays (22). Those sub-ps datasets demonstrate oscillatory signals at frequencies around 100 cm^–1^ (see below). But no signal of conformational change, other than the extensive oscillations, is found from these data in the transmembrane helices or the chromophore depicting the retinal isomerization despite the original report. The essence of this work is a numerical resolution of structural heterogeneity, a common difficulty often encountered in cryo trapping and time-resolved crystallography. To what extend a specific structural species can be enriched in crystals depends on the reaction kinetics governed by many experimental parameters including but not limited to the fluence, wavelength, and temperature of the light illumination. While it is possible to reach higher fractional concentrations at specific time points for more stable species such as K and M states of bR due to the ratio between the rates going into and exiting from that species, transient species such as I and J are often poorly populated. If such structural heterogeneity is not resolved, it is very difficult, if not impossible, to interpret the electron density maps and to refine the intermediate structures (23). An assumption in nearly all previous studies has been that each dataset, at a cryo temperature or at a time delay, is derived from a mixture of a single conformer of a photoinduced species and the ground state. Therefore, the difference map reveals a pure intermediate structure. This assumption is far from the reality, thus often leads to misinterpretation of the observed electron density maps. This work is yet another case study to demonstrate the application of our analytical protocol based on SVD (see the “Methods” section) that makes no assumption on how many excited intermediates that contribute to the captured signals at each time point (23–25). More importantly, this work showcases that our resolution of structural heterogeneity enables new mechanistic insights into the highly dynamic chemical or biochemical processes.

## Results and discussion

**Fig. 1.**
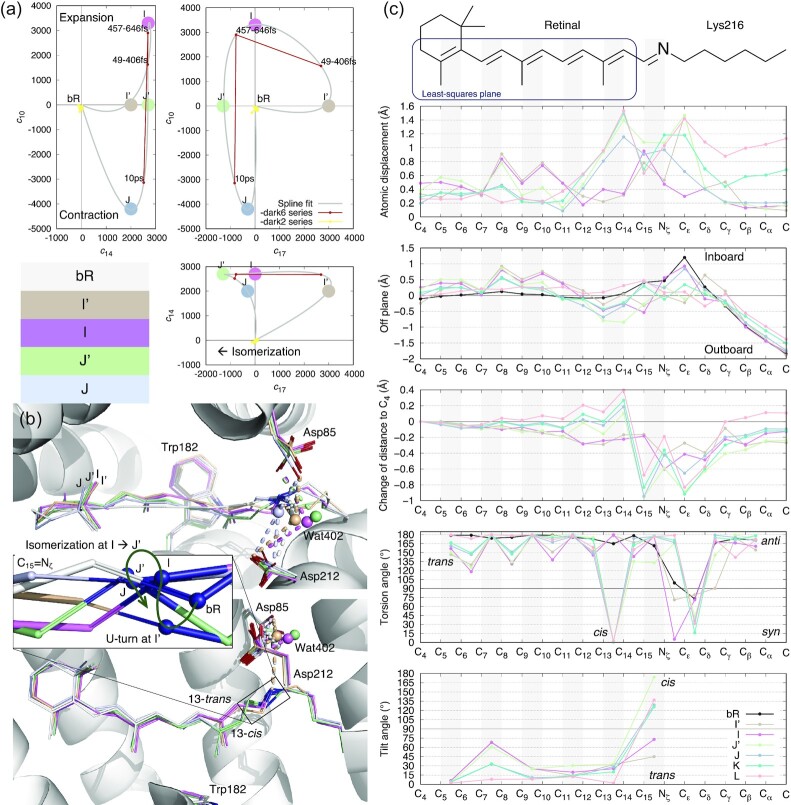
Intermediate species in early photocycle identified in SVD space. (a) Multi-dimensional spaces of SVD. The SVD analysis of difference Fourier maps at short delays ≤10 ps results in time-dependent coefficients *c_k_*(*t*), where *k* = 1, 2, . . ., each corresponding to a time-independent components ***U**_k_*. Each raw difference map at a time delay *t* can be closely represented by a linear combination of these components, *c*_1_(*t*)***U***_1_ + *c*_2_(*t*)***U***_2_ + . . ., that is called a reconstituted difference map. Each of these components ***U****_k_* and the reconstituted difference maps can be rendered in the same way as an observed difference map. The coefficient set *c_k_*(*t*) is, therefore, a trace of the photocycle trajectory, when these time-dependent functions are plotted in a multi-dimensional space or plotted together against the common variable of time *t*. Coefficients corresponding to components ***U***_10_, ***U***_14_, and ***U***_17_ are plotted in three orthographical views. Three time points from Nogly et al. in small red dots contain ***U***_14_ equally. These time points vary in ***U***_10_ and ***U***_17_. Datasets from Kovacs et al. in small yellow dots do not carry any of these signals, therefore cluster near the origin. The component map of ***U***_10_ is displayed in Fig. 3c. ***U***_14_ is displayed in Figs 2a and Supplementary S5. ***U***_17_ is displayed in Fig. 2b. Several apices of the spline fitting are chosen as the potential pure states of I′, I, J′, and J marked by large dots. This choice is only an approximate due to the insufficient number of time points observed. (b) The refined retinal conformations compared with the resting state in white. I′, I, J′, and J are in beige, purple, green, and bluish gray, respectively. The creased S-shape of the retinal is established in I′ (Fig. 2) and easing gradually (Fig. 1b and c). 13-*trans* in I′ and I, 13-*cis* in J′ and J are two discrete conformations without any other conformations in the middle (c, fourth panel). The inset is a zoom-in view of the SB double bond C_15_=N_ζ_. The SB N_ζ_ atom performs two rapid U-turns at ∼30 and ∼500 fs. (c) Conformational parameters calculated from the refined chromophore. The chemical structure of the chromophore on top is aligned to the horizontal axis. Double bonds are shaded in gray. Intermediates K and L are taken from the companion paper (19). (1) Atomic displacements of each intermediate from the resting state show greater changes in the proximal segment around the SB (top panel). See Supplementary Fig. S1 for definitions of proximal, distal, inboard, outboard, and other orientations in bR molecule. (2) A plane is least-squares fitted to C_4_ through C_14_ of the resting state as boxed on the chemical structure. The distances of all atoms to this plane in the inboard and outboard directions show the curvature of the chromophore. The creased retinal in early intermediates and the inboard protruding corner at C_ε_ in the resting state are clearly shown (second panel). (3) Distances to atom C_4_ are calculated for all refined chromophores. Changes in these distances with respect to the resting state show the shortened chromophore in I′ and I. Once isomerization to 13-*cis* occurs, the segment from C_15_ to C_δ_ around the SB becomes significantly closer to the β-ionone ring due to the Coulombic attraction force, while the distal segment of the retinal from C_14_ and beyond stretches (third panel). (4) The torsion angles of single and double bonds quantify *anti*/*syn* or *trans*/*cis* for the ground state and all intermediates (fourth panel). (5) Only a single bond can be twisted with its torsion angle near 90°. A twisted double bond would be energetically costly. Each double bond is least-squares fitted with a plane. The interplanar angle between a double bond and the corresponding one in the ground state measures the local tilting of the retinal (bottom panel).

A total of 22 datasets and 18 time points up to 10 ps are analyzed in this study (Supplementary Table S1). Difference Fourier maps at different time points and with respect to their corresponding dark datasets are calculated according to the protocols previously described (see the “Methods” section). A collection of 126 difference maps at short delays ≤10 ps are subjected to SVD followed by a numerical deconvolution using the previously established Ren rotation in a multi-dimensional Euclidean space (24, 26). Such resolution of electron density changes from mixed photoexcited species in the time-resolved datasets results in four distinct intermediate structures in the early photocycle (Fig. 1), which are then refined against the reconstituted structure factor amplitudes (Supplementary Table S2; see the “Methods” section).

### Low-frequency oscillations upon photoexcitation

Out of 17 major components derived from the sub-ps delays of Kovacs et al. (Supplementary Fig. S2c), 10 components describe five two-dimensional oscillatory behaviors at frequencies ranging from 60 to 400 cm^–1^ (Supplementary Fig. S3). Compared to a bond stretching frequency commonly observed in vibrational spectroscopy, these oscillations are at much lower frequencies. The lowest frequency is 61 ± 2 cm^–1^, that is, a period of 550 ± 20 fs (Supplementary Fig. S3a), observed for more than an entire period, which matches exactly the oscillation detected in transient absorption changes in visual rhodopsin (27). The second lowest frequency at 150 ± 3 cm^–1^ is observed for more than one and a half periods, and the highest frequency oscillation is observed for nearly six periods at 396 ± 3 cm^–1^ (Supplementary Fig. S3). Although these ten components follow the oscillatory time dependencies nicely, they do not show any association with the chromophore or any secondary structure of the protein (Supplementary Fig. S4). Similar oscillatory components were also extracted from the time-resolved XFEL datasets of carbonmonoxy myoglobin (24). Therefore, the same conclusion stands, that is, these low-frequency vibrations induced by short laser pulses often detected by ultrafast spectroscopy are the intrinsic property of a solvated protein molecule (28, 29). However, the functional relevance of these oscillations is unclear. Interestingly, the isomerization sampling and productive photoisomerization observed in this study occur within the first oscillatory period at the lowest frequency. While such coincidence begs the question whether the protein oscillation is required for isomerization (see below), direct evidence is lacking in these XFEL data to support any functional relevance of these oscillatory signals.

These oscillations originate solely from the sub-ps datasets (Supplementary Fig. S2ab yellow, green, blue, and purple traces) of Kovacs et al. derived from excessive peak power density of their pump laser (22, 30). The datasets of Nogly et al. in the same delay range carry coefficients ≈ 0 corresponding to these oscillatory components, that is, free of oscillation (Supplementary Fig. S2ab red and brown traces near the origin). On the other hand, no dataset of Kovacs et al. features significantly nonzero coefficient other than those corresponding to the oscillatory components (Fig. 1a yellow trace near the origin). Therefore, these datasets containing oscillations do not carry any interpretable signal in the protein or on the chromophore, at least not in the outstanding components identified by SVD (Supplementary Fig. S2c). As Miller et al. pointed out, it is entirely possible that the specimens were pumped onto higher potential energy surfaces due to multiphoton absorption and the subsequent decays follow some biologically irrelevant reaction pathways (30).

### Intermediates I′ and I, and expansion of retinal binding pocket

**Fig. 2.**
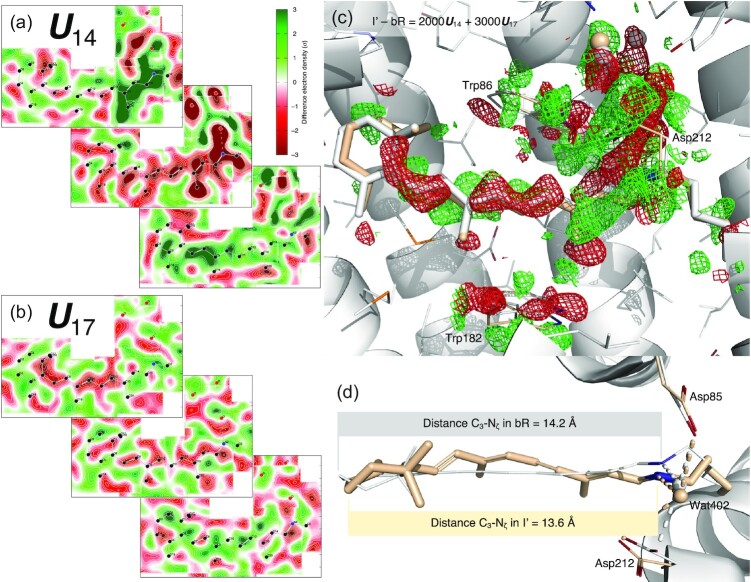
Shortened retinal in S-shape since earliest intermediate I′. (a) Cross-sections of component map ***U***_14_. The middle cross-section is an integration ±0.2 Å around the curved surface through the retinal. The top cross-section is an integration 1.2 to 1.8 Å outboard from the retinal surface and the bottom one is an integration 0.8–1.2 Å inboard. See Supplementary Fig. S1 for definitions of inboard, outboard, proximal, distal, and other orientations in bR molecule. Green and red indicate electron density gain and loss, respectively. Nearly the entire retinal is in negative densities. The proximal segment and three waters are in intense negative densities. On the other hand, strong positive densities flank the proximal and distal segments from the outboard and inboard, respectively. Such signal distribution results in the S-shaped retinal by the refinement shown in (d). (b) Cross-sections of component map ***U***_17_. The middle cross-section is an integration ± 0.2 Å around the surface through the retinal. The top panel is an integration 0.5 to 0.9 Å outboard and the bottom is an integration 0.8 to 1.2 Å inboard. Negative and positive densities flank the retinal from the outboard and inboard, respectively. (c) Difference map of I′− bR reconstituted from ***U***_14_ and ***U***_17_ (a and b). The map is contoured at ±3*σ* in green and red mesh, respectively. The opposite displacements of the distal and proximal segments of the retinal are obvious. Extensive signals indicate changes in the water network and Asp85 and 212. (d) Refined retinal conformation in beige overlaid on the resting state in white. This view is orthographical to (c). The marked distances from C_3_ to N_ζ_ show a shortened retinal creased into an S-shape. C_20_ methyl group is tilted 33° toward outboard from its resting state bR (Fig. 1c, bottom panel). Wat402 remains in H-bonds with both Asp85 and 212.

**Fig. 3.**
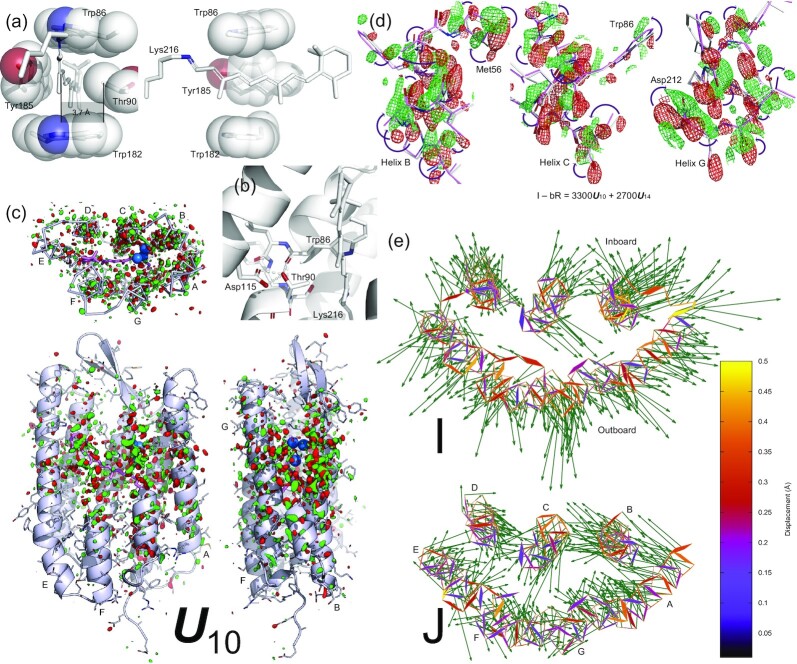
Retinal binding pocket expansion and contraction. (a) Two orthographical views of the retinal tightly boxed at its middle segment. The closest contact is Thr90 and Tyr185 on the inboard and outboard sides of the retinal plane, respectively. The minimum distance between them is 7.0 Å = 4*r*_C_ + 0.2 Å, where *r*_C_ = 1.7 Å is the van der Waals radius of C atom. The C_γ_ methyl group is 3.7 Å = 2*r*_C_ + 0.3 Å away from the retinal plane. See also (2). (b) H-bond network involving Thr90. The O_γ_ hydroxyl group of Thr90 is strongly H-bonded with the main chain carbonyl of Trp86 in helix C and the carboxyl group of Asp115, simultaneously. These H-bonds fixate the side chain of Thr90 so that the C_γ_ methyl group is pointing to the retinal plane directly. (c) Component map ***U***_10_. The main chain and side chains of the protein are rendered with ribbons and/or sticks. The retinal and Lys216 are in purple sticks. Several key waters are in blue spheres. Parts of the structure are omitted to reveal more of the interior. The map is contoured at ±3*σ* in green and red, respectively. Three orthographical views of ***U***_10_ clearly show that the signals are distributed around the middle segment of the molecule and taper off to both cytoplasmic and extracellular surfaces. The signals also concentrate on all seven helices. (d) Reconstituted difference map I − bR from ***U***_10_ (c) and ***U***_14_ (Figs 2a and Supplementary S5). The map is contoured at ±2.5*σ* in green and red mesh, respectively. The difference map at three middle segments of helices B, C, and G show main chain displacements toward inboard or outboard as indicated by the arrows marking the negative and positive pairs of densities. These difference densities are the direct evidence of the expansion of the retinal binding pocket. The refined structure of I is in purple, and the resting state is in white. (e) The refined structures of I and J compared with the resting state viewed along the trimer three-fold axis from the extracellular side. Atomic displacements in the main chain from bR to I and J are color coded and marked by arrows with lengths 20 × of the actual displacements. All seven helices in I move away from the center except a small segment in helix C showing an expansion of the retinal binding pocket (top panel). However, all seven helices in J move closer to one another showing a contraction with respect to the resting state bR (bottom panel). This contraction is much more significant if compared directly with the expanded I state.

In contrast to the oscillating signals, three components ***U***_10_, ***U***_14_, and ***U***_17_ reveal strong light-induced structural signals in terms of both extensiveness and quality (Fig. 2a, b and Supplementary S5). These signals originate exclusively from a few time points of Nogly et al., too few to fit the time dependency with exponentials. Instead, a spline fitting through these time points gives rise to the estimated coefficients *c*_10_, *c*_14_, and *c*_17_ in the linear combination of *c*_10_***U***_10_ + *c*_14_***U***_14_ + *c*_17_***U***_17_ for reconstructing the electron density maps of the states I, J, and their respective precursors I′, J′ (Fig. 1a). A reconstituted difference map of I′ − bR = 2000***U***_14_ + 3000***U***_17_ (Fig. 2c) is located on the spline trajectory from the origin, that is, bR at the time point of 0-, to the first time point of 49 to 406 fs (PDB entry 6g7i). This state is denoted I′ as a precursor leading to the I state judged by the time point at ∼30 fs. However, this is not to say that a single species I′ exists around 30 fs. Quite the opposite, the population of the time-independent conformational species I′ rises and falls and peaks around 30 fs, while many other species during isomerization sampling coexist with I′ at the same time (see below). The reconstituted difference map is used to calculate a set of structure factor amplitudes that would produce this difference map of I′ − bR (see the “Methods” section). And the structure of I′ is refined against this reconstituted dataset (beige; Figs 2c, d and Supplementary S6). The same protocol is used to refine the structure of I state (purple; Supplementary Fig. S7) with a reconstituted difference map I − bR = 3300***U***_10_ + 2700***U***_14_ (Fig. 3d). This SVD-dependent refinement strategy extends the commonly used method based on an extrapolated map to another level. This newly developed method is able to refine a structure against any linear combination of signal components while eliminating noise and systematic error components, and components identified as other intermediate species mixed in the data. Therefore, this method enables the refinement of an unscrambled, hence pure, structural species (see the “Methods” section).

Five geometric parameters are calculated along the long-chain chromophore in the refined intermediate species to depict the transient conformation (Fig. 1c): (1) Atomic displacements along the polyene chain and the lysyl side chain of Lys216 with respect to the ground state structure demonstrate the overall amplitude of conformational change of the chromophore (Fig. 1c, top panel). (2) The retinal in the ground state is largely flat except C_15_ and the methyl groups of C_16_ and C_17_ (Supplementary Fig. S1e). Five consecutive double bonds from C_5_ to C_14_ of the retinal are largely coplanar. Atoms along the polyene chain and the lysyl side chain of Lys216 are located on both sides of this retinal plane. The side toward the three-fold axis of the bR trimer is called inboard; the opposite side is outboard (see Supplementary Fig. S1 for orientations in bR molecule). Distances from the atoms along the long-chain chromophore to the retinal plane in the ground state are calculated for all intermediates (Fig. 1c, second panel). This parameter shows the curvature of the refined conformation. (3) Changes in the distances from the atoms along the chromophore to C_4_ show extension or contraction of the chromophore in various intermediates (Fig. 1c, third panel). (4) Torsion angles of the single and double bonds along the chromophore clearly demonstrate *syn*/*anti* and *cis*/*trans* conformation as well as possible deviation from the ideal bond geometry in the refined intermediates (Fig. 1c, fourth panel). (5) Six double bonds of the chromophore are refined to either *cis* or *trans* configurations in the ground state and all intermediates with small deviations from the ideal geometry. Therefore, each double bond can be fitted with a plane. The fitted plane of each double bond tilts from the ground state as the conformation evolves through the intermediates (Fig. 1c, bottom panel). These conformational parameters provide a good metric to evaluate and compare the intermediate species.

In contrast to the flat all-*trans* retinal chromophore in the ground state (Fig. 1c, second panel), the side chain of Lys216 is highly twisted forming two near-90° single bonds (Fig. 1c, fourth panel), which results in a corner at C_ε_ that deviates dramatically from the plane of the all-*trans* retinal (Fig. 1c, second panel). This corner and its interaction with helix C is important to the function of this proton pump (19), which is a topic outside the scope of isomerization sampling here. The refined geometry of the retinal in I′ retains a near perfect all-*trans* configuration, including the SB double bond C_15_=N_ζ_, while various single bonds along the polyene chain deviate from the standard *anti* conformation significantly (Fig. 1c, fourth panel). The torsional deviations from *anti* are in a descending order from the β-ionone ring to the SB. However, the largest torsional twist to 90° is found at the single bond C_δ_−C_γ_ in the Lys216 anchor (Fig. 1c, fourth panel). These torsional changes at single bonds are among the earliest conformational responses to photon absorption due to the low cost in energy. On the contrary, no immediate change is visible at the double bonds (see below). The torsional changes of the single bonds result in an S-shaped retinal shortened by ∼4% (Fig. 1c, third panel). The distal segment C_6_−C_12_ moves inboard up to 0.9 Å and the proximal segment C_13_−C_ε_, including the SB, moves outboard up to 1.6 Å (Fig. 1c, first and second panels). This creased retinal observed here at around 30 fs (Fig. 2d) is attributed to the direct consequence of a compression under an attraction force between the β-ionone ring and the SB (see below).

The refined structure of the I state (Supplementary Fig. S7) shows that the retinal remains in near perfect all-*trans*, including the SB, and as creased as its precursor I′ (Fig. 1d). The torsional deviations from *anti* single bonds become even more severe compared to the I′ state and remain in a descending order from the β-ionone ring to the SB (Fig. 1c, fourth panel). The major difference from its precursor is that the single bond N_ζ_−C_ε_ now adopts a perfect *syn* conformation, and C_δ_–C_γ_ is restored to perfect *anti* (Figs 1c, fourth panel and 3c). As a result, the anchor Lys216 has largely returned to its resting conformation. This prompt restoration of the resting conformation is found important to the pumping mechanism of bR presented in the companion paper (19). Such a lack of substantial change between the ground state and the intermediate I was previously noted by a comparison of the native retinal to a chemically locked C_13_=C_14_ double bond (16).

Remarkably, the major component ***U***_10_ reconstituted into the difference map of I − bR contains widespread signal associated with all seven helices (Fig. 3c). The reconstituted map clearly shows collective outward motions from the center (Fig. 3d) suggesting an expansion of the retinal binding pocket at hundreds of fs, which is confirmed by the refined structure of the I state (Fig. 3e, top panel). For example, the distances between the C_α_ atoms increase by 0.8 Å between Arg82 and Phe208 and by 0.7 Å between Tyr83 and Trp182. It is noteworthy that similar signals in the protein are present in the raw difference map calculated from the time point of 457 to 646 fs from Nogly et al. (6g7j) prior to an SVD analysis (Supplementary Fig. S8). The refined structure also shows the expansion to a lesser extent (Supplementary Fig. S9, top panel). But this expansion of the retinal binding pocket was not reported (21).

Transient bleaching at near UV of 265–280 nm was observed before 200 fs and attributed to structural changes in the retinal skeleton and the surrounding Trp residues (31). Recent deep-UV stimulated Raman spectroscopy also demonstrated that motions of Trp and Tyr residues start to emerge at 200 fs and remain steady until the isomerization is over at 30 ps (17). Here, the refined structure of the I state with displaced helices and an expanded retinal binding pocket offers an explanation for the stimulated Raman gain change at hundreds of fs. However, it is unclear why and how such extensive protein responses take place even before the retinal isomerization. According to the broadly accepted concept of proteinquake, initial motions are generated at the epicenter where the chromophore absorbs a photon and then propagated throughout the protein matrix (32). It is plausible that these ultrafast protein responses are the direct consequence of isomerization sampling in a confined protein pocket. It was observed in organic solvents using high-pressure liquid chromatography (HPLC) that all-*trans* retinal could isomerize at various double bonds along the polyene chain to adopt 9-, 11-, and 13-*cis* configurations, but with rather poor quantum yields (8, 9). This intrinsic property of the all-*trans* retinal would behave the same even when it is incorporated in the protein except that the protein matrix herds the chromophores on the right track of the productive photocycle and keeps the concentrations of the attempted byproducts low. These byproduct conformations of the retinal during isomerization sampling are too numerous and too minor to be observed experimentally. Nevertheless, they cause a common effect, an expansion of its binding pocket, since the all-*trans* retinal in the resting state is tightly boxed by massive side chains all around (Figs 3a and Supplementary S10). Any attempt to isomerize would push against this box one way or another. For instance, triple attempts to isomerize simultaneously at 11, 13, and 15 positions were suggested by a quantum mechanics/molecular mechanics simulation (33). A molecular dynamics simulation also concluded that the protein arrests many different photoisomerization paths and funnels them into a single, productive path (34). When the retinal binding pocket is altered in mutants, the quantum yield of each isomerization byproduct is expected to increase resulting in an impaired productive pathway (see below).

### Intermediates J′ and J, and productive isomerization of retinal

The time point of 10 ps of Nogly et al. (6g7k) differs from the previous time point of 457 to 646 fs (6g7j) by negating the component of ***U***_10_ (Fig. 1a), which leads to a restoration of the normal retinal binding pocket in J′ from an expanded one in the I state followed by a contraction in J (Fig. 3e, bottom panel). Two time-independent structures of J′ (green; Supplementary Fig. S11) and J (gray; Supplementary Fig. S12) are refined with the same protocol based on the respective reconstituted difference maps J′ − bR = 2700***U***_14_ – 1300***U***_17_ and J − bR = −4200***U***_10_ + 2000***U***_14_– 300***U***_17_ (see the “Methods” section). Their populations peak at the approximate time of ∼700 fs and ∼20 ps, respectively. The observed contraction of the retinal binding pocket is likely due to an elastic recoiling of the seven helical bundle following its transient expansion caused by the isomerization sampling. A slight contraction is also present in the refined structure of 6g7k at 10 ps (Supplementary Fig. S9, bottom panel).

**Fig. 4.**
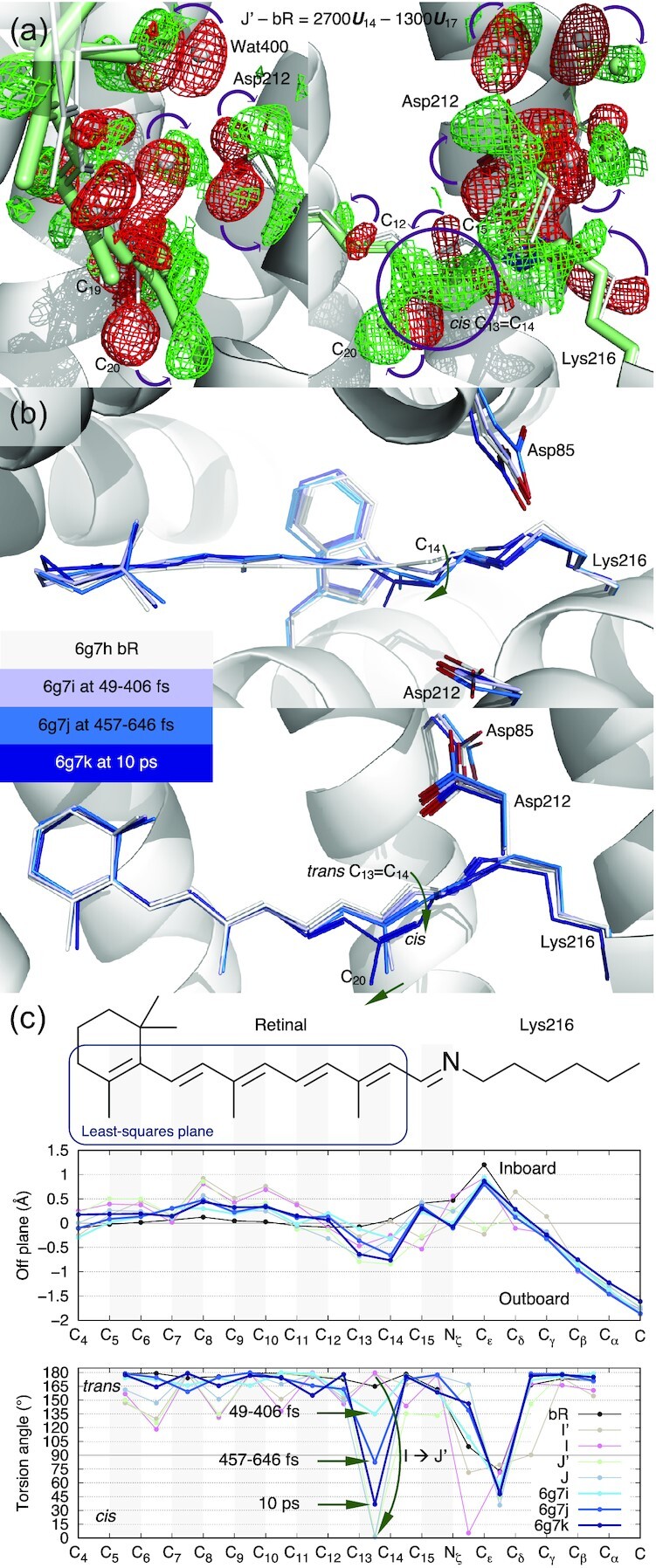
Photoisomerization at C_13_=C_14_ of retinal in bR. (a) Reconstituted difference map J′− bR from ***U***_14_ and ***U***_17_ (Figs 2a, b and Supplementary S5). The map is contoured at ±3.5*σ* in green and red mesh, respectively. These difference densities are the direct evidence of isomerization at hundreds of fs. The refined structure of J′ in 13-*cis* is in green. C_13_=C_14_ in perfect *cis* is shown in the circled green positive density. J′ is the earliest 13-*cis* species at hundreds of fs. (b) Published structures of Nogly et al. The chromophore conformations in darker and darker blues are overlayed with the resting state in white. Photoisomerization is interpreted as a gradual change in conformation from tens to hundreds of fs to ps. Even at 10 ps, C_13_=C_14_ is not yet in perfect *cis* (21). (c) Conformational parameters calculated from the published structures of Nogly et al. The chemical structure of the chromophore on top is aligned to the horizontal axis. Double bonds are shaded in gray. (1) A plane is least-squares fitted to C_4_ through C_14_ of the resting state as boxed on the chemical structure. The distances of all atoms to this plane in the inboard and outboard directions show slight crease of the chromophore captured by the structures of Nogly et al. (top panel). (2) The torsion angles indicate no significant rotation of single bonds captured by the structures of Nogly et al. (bottom panel). However, C_13_=C_14_ is transitioning gradually from *trans* to *cis*. In contrast, the refined structures of I and J′ show a complete photoisomerization from perfect *trans* to perfect *cis* directly.

The creased retinal persists in J′ but starts to relax back to a flat conformation in J (Fig. 1b and c, second panel). The difference map of J′ − bR clearly shows the 13-*cis* configuration (Fig. 4a). Indeed, near perfect 13-*cis* is successfully refined in both J′ and J structures (Fig. 1c, fourth panel). While the SB double bond C_15_=N_ζ_ is momentarily distorted from the *trans* configuration in J′ with a torsion angle of 133°, a perfect *trans* configuration at C_15_=N_ζ_ is promptly restored in J (Fig. 1c, fourth panel). The refined structures of this series of early intermediates show that the SB N_ζ_ is rotating clockwise in the entire process of the isomerization of I′ → I → J′ → J, if the retinal is viewed from the proximal to distal direction. It seems that the isomerization starts in an expanded retinal binding pocket and finishes in a tighter one. Whether the pocket expansion and contraction are required for the productive isomerization and what role the low-frequency oscillations play in isomerization, will need more time points at short delays to further isolate the molecular events temporally.

The previous interpretations of the mixed signals of multiple intermediate species presented a rotation of the C_14_ atom, instead of N_ζ_, in the counterclockwise direction if viewed from the proximal end of the retinal (Fig. 4b) (22, 21). This rotation starts at the time point of 49 to 406 fs (6g7i) and proceeds continuously through hundreds of fs to ps. At 10 ps time point (6g7k), this rotation still does not complete and results in the remaining twist of 37° in the torsion angle of C_13_=C_14_ (Fig. 4c, bottom panel). An experimentally determined double bond with a torsion angle of 82°, for example 6g7j, indicates that this twisted double bond conformation is more populated than any other conformation at hundreds of fs. Such interpretation is in direct contradiction of the current view in molecular dynamics. In theory, a double bond conformation spends most time in either energy well of *trans* or *cis*. The transition time between these energy wells is relatively short. A twisted double bond conformation cannot be significantly populated compared to the discrete populations in *trans* and *cis*, that is, cannot be observed, unless an additional energy well emerges near the torsion angle of 90°, which no evidence supports. The gradual rotation of C_13_=C_14_ double bond presented in the refined structures of Nogly et al. and Kovacs et al. was a misinterpretation of the mixed signals of multiple intermediate species as a single conformer. The gradual rotation does not occur in isomerization, rather it merely reflects the gradual population shift from discrete states of *trans* to *cis*. In stark contrast, this work presents that perfect *trans* to perfect *cis* isomerization occurs in a single move during the transition of I → J′ (Fig. 4c, bottom panel). Are these two different interpretations of the same data equivalent and inconsequential to mechanistic understanding? The discrete states in *trans* and *cis* presented here result in two sharp U-turns of the SB N_ζ_ not observed in the previous interpretations, the first one at I′ around 30 fs and the second one during I → J′ isomerization at ∼500 fs (Fig. 1b inset). The functional implication of these ultrafast SB U-turns is presented in the companion paper (19).

### Coulombic attraction as driving force of isomerization sampling

The fundamental questions remain: What is the driving force that causes the all-*trans* retinal to isomerize after a photon absorption, at several double bonds with a preference at C_11_=C_12_ if not restrained but exclusively at C_13_=C_14_ in bR? Which structural feature(s) of the chromophore and/or its protein environment differentiate the excited state from the ground state? How does the protein environment enhance the quantum yield and speed up the reaction rate of the isomerization to 13-*cis* by tuning the shape of the potential energy surfaces? When does the crossing of the conical intersection occur along the photocycle trajectory? Here, I hypothesize that a Coulombic attraction between the β-ionone ring and the SB at the Frank–Condon point, 0+time point, provides the initial driving force upon a photon absorption. The electric field spectral measurements (35) and the quantum mechanics simulation (21) suggested that a charge separation occurs along the polyene chain at the excited state of bR. Such a dipole moment was also detected through a transient bleaching signal at near UV region (31). It can be shown that a plausible charge separation of ±0.1e between the β-ionone ring and the SB would cause an attraction force > 1 pN. If calibrated with the measured range of dipole moment of 10 to 16 D (35), the charge separation could reach the level of ±0.16e to ±0.26e, giving rise to an attraction force of 3.5 to 9 pN between the β-ionone ring and the SB. This attraction force is evidently sufficient to crease the flat all-*trans* retinal into an S-shape and to compress it slightly within tens of fs as observed in I′ and I states [Fig. 1b and c (second and third panels) and Fig. 2d]. In the meanwhile, this very attraction force also triggers simultaneous attempts of double bond isomerizations and single bond rotations along the polyene chain that cause the expansion of the retinal binding pocket as observed at hundreds of fs. Despite such observable expansion, the elastic strength of the seven helical bundle blocks all reaction pathways of the isomerization sampling except the productive one. Following the only successful isomerization at C_13_=C_14_, the chromophore segment from C_15_ to C_δ_ is attracted to the β-ionone ring; and these two parts become significantly closer (Fig. 1c, third panel). None of the single bond rotations can complete under the restraints of the protein (Figs 3a and Supplementary S10). Especially, the segment closer to the midpoint of the retinal is more confined due to the steric hinderance of Thr90 and Tyr185 from the inboard and outboard sides, respectively (Fig. 3a). Therefore, the single bonds deviate from *anti* less and less towards the midpoint (Fig. 1c, fourth panel).

I could further speculate that the charge separation along the polyene chain differentiates the excited state from the ground state. Therefore, the disappearance of any structural effects caused by the charge separation marks the crossing of the conical intersection. The light-induced charge separation seems to subside gradually as the reaction proceeds beyond the K state as indicated by the slow restoration of the perfect *anti* conformation and the reluctant flattening of the retinal plane (Fig. 1c, second and fourth panel), where the refined structures of K and L are taken from the companion paper (19). If it is safe to assume that the single bond distortion away from a perfect *anti* and the extent of the crease in the retinal plane are direct measures of the charge separation with a delay no longer than ns time scale, the observed crossing of the conical intersection is surprisingly late during the K → L transition at a few μs. The structural basis of tuning potential energy surface arises from the stereochemical hinderance of the retinal binding pocket that makes all isomerization attempts and single bond rotations energetically costly except one.

Apparently, the same charge separation and the attraction force upon photon absorption also take place in a solution sample of free retinal. Compared to the retinal embedded in protein, photoisomerization in solution is nonspecific, resulting in a range of byproducts, since an isomerization at any position would bring the SB significantly closer to the β-ionone ring. The major photoproduct is 11-*cis* among several possibilities in solution simply because C_11_=C_12_ double bond is located at the midpoint from the β-ionone ring to the SB, thus, most vulnerable to a mechanical twist. It is also understandable that each of the byproducts could only achieve a poor quantum yield (8, 9) as rotations at multiple single bonds driven by the same attraction force and achieving a similar kink in the polyene chain would further sidetrack the double bond isomerizations thus diminishing their quantum yields. Since the total quantum yield of isomerization at various double bonds is ∼1/4, it can be predicted that the total quantum yield of single bond rotations is ∼3/4 in solution. Because the deeper valleys on the potential energy surface leading to single bonds in *syn* are more energetically favorable routes than the shallower valleys leading to *cis* double bonds. However, these byproducts due to single bond rotations are short-lived beyond detection by HPLC as they spontaneously revert back in solution. The protein environment in bR plays a major role in enhancing the quantum yield and accelerating the reaction rate of the isomerization to 13-*cis* by shutting down all other reaction pathways triggered by the same charge separation. This is further elucidated by the mutant functions below.

### Isomerization byproducts permitted by mutant protein environments

The structure of a double mutant T90A/D115A (3cod) showed little difference from the wildtype (36), while the single mutants T90V and T90A retain < 70% and < 20% of the proton pumping activity, respectively (37, 38). These observations illustrate that some nonproductive pathways of the isomerization sampling succeed more in the altered retinal binding pocket. In the wildtype structure, Thr90 in helix C points towards the C_11_=C_12_−C_13_−C_20_ segment of the retinal from the inboard with its C_γ_ atom 3.7 Å from the retinal plane (Fig. 3a). Given the van der Waals radius *r*_C_ of 1.7 Å, only 0.3 Å is spared for the H atoms of the C_γ_ methyl group, thereby effectively shutting down the nonproductive pathways of the isomerization sampling. In addition, the C_ε_ methyl group of Met118 and the C_δ_ ethyl group of Ile119 in helix D are 3.7 to 4.6 Å away from the retinal segment of C_7_=C_8_−C_9_=C_10_ and C_19_ from the inboard (Supplementary Fig. S10). Any motion of the retinal would have to push helices C and D toward inboard causing an expansion of its binding pocket. Missing the close contact in T90A increases the room to 1.9 Å for isomerization byproducts, which would greatly reduce the quantum yield of the 13-*cis* productive isomerization thus retain < 20% of the activity.

In addition to 13-*cis*, the retinal in the light adapted T90V mutant showed 9- and 11-*cis* configurations at the occupancies of 3% and 18%, respectively, while these configurations were not detected in light adapted wildtype (37). Then why would a Val residue at this position with an equivalent C_γ_ methyl group permit the formation of some isomerization byproducts? In wildtype bR, the side chain of Thr90 engages two strong H-bonds Trp86O−Thr90O_γ_−D115O_δ_ so that its C_γ_ methyl group is aligned toward the retinal (Fig. 3b). Without these H-bonds in T90V, the isopropyl group of Val90 is free to adopt other rotameric positions so that neither of the C_γ_ methyl groups has to point directly to the retinal, which increases the available room for the formation of some isomerization byproducts. The conformational change caused by the Val substitution at this position was detected by spectroscopy (39). Compared to the light adapted state with 3% 9-*cis* and 18% 11-*cis*, these isomerization byproducts could reach even higher percentages during active photocycles thus reduce the proton pumping activity below 70%.

It is important that the C_γ_ methyl group of Thr90 in the wildtype is pointing to C_11_=C_12_ double bond located at the midpoint of the retinal as 11-*cis* is the most probable byproduct (6, 7). The potential energy surface of the excited state in solution must feature a major branch of energetic valley leading to 11-*cis*. But this valley is blocked by the stereo hinderance of Thr90 braced by the H-bonds.

From the outboard, the side chain of Tyr185 in helix F is nearly parallel to the mid segment of the retinal plane with a distance of 3.5 Å (Fig. 3a), and Pro186 is also parallel to the distal segment with 3.7 to 4.4 Å distance (Supplementary Fig. S10). These close contacts of flat areas from C_5_ to C_14_ of the retinal prevents any significant motion of the retinal toward the outboard. Even slight motions would push helix F away as observed here in the expansion of the retinal binding pocket. The mutant Y185F largely retains the flat contact so that its proton pumping activity does not reduce much (40, 41). However, it is predictable that various single mutants at this position with smaller and smaller side chains would promote more and more isomerization byproducts, and eventually shut down proton pumping.

Two massive side chains of Trp86 and 182 from the extracellular and cytoplasmic sides, respectively, do not seem to play a significant role in suppressing byproduct formation as shown by the mutant W182F that retains the most of the wildtype activity (40), since the motions involved in isomerization sampling are oriented more laterally. The transient expansion and contraction of the retinal binding pocket (Fig. 3e) indicate that the tight box surrounds the mid-segment of the retinal (Fig. 3a) is not completely rigid. Rather, its plasticity must carry sufficient strength to prevent isomerization byproducts. Presumably, this strength originates from the mechanical property of the helical bundle.

## Concluding remarks

In summary, this work employs a numerical resolution from concurrent structural events, hence substantially improves the quality of electron density maps of unscrambled intermediate species that are otherwise difficult to observe. This approach reveals the transient structural responses to many unsuccessful attempts of double bond isomerization and single bond rotation. These findings underscore an important implication, that is, a nonspecific Coulombic attraction provides the same driving force for the isomerization sampling with and without a protein matrix. A productive isomerization at a specific double bond is guided by the incorporation of the chromophore in a specific protein environment. The productive pathway is selected from numerous possibilities via stereochemical hinderance. This case study offers a detailed example elucidating how a protein structure achieves regioselectivity in a biochemical reaction that catalyzes a specific double bond isomerization by accelerating its reaction rate and shutting down all other byproduct pathways. Nevertheless, this nonspecific Coulombic attraction force may not be directly applicable to the photoisomerization of retinal from 11-*cis* to all-*trans* in the activation of visual rhodopsins. The commonality among microbial and animal rhodopsins perhaps exists only at a high level in a rather abstract sense, if any. These photoreactions to and from the all-*trans* retinal not necessarily share any common trajectory on the potential energy surfaces. The key difference is bR as an energy convertor versus a visual rhodopsin as a quantum detector (42).

## Supplementary Material

pgac103_Supplemental_File

## Data Availability

The structures are deposited to PDB-Dev (pdb-dev.wwpdb.org) with accession codes PDBDEV_00000129, 138, 139, and 140, respectively.

## Notes

**Competing interest:** ZR is the founder of Renz Research, Inc., which currently holds the copyright of the computer software dynamiX™.
