# Supplementary material for: Photoinduced isomerization sampling of retinal in bacteriorhodopsin
Source: PNAS Nexus. 2022 Jul 1;1(3):pgac103. doi: 10.1093/pnasnexus/pgac103 (PMC9364214; doi:10.1093/pnasnexus/pgac103)
Supplement: pgac103_Supplemental_File [file pgac103_supplemental_file.pdf]

Photoinduced Isomerization Sampling of Retinal in Bacteriorhodopsin

Zhong Ren

Department of Chemistry, University of Illinois at Chicago, Chicago, IL 60607, USA
Renz Research, Inc., Westmont, IL 60559, USA

zren@uic.edu
ORCID 0000-0001-7098-3127

Methods

From the outset, the key presumption is that every crystallographic dataset, at a given temperature and a given time delay after the triggering of a photochemical reaction, captures a mixture of unknown number of intermediate species at unknown fractions. Needless to say, all structures of the intermediates are also unknown except the structure at the ground state that has been determined and well refined by static crystallography. A simultaneous solution of all these unknowns requires multiple datasets that are collected at various temperatures or time delays so that a common set of intermediate structures are present in these datasets with variable ratios. If the number of available datasets is far greater than the number of unknowns, a linear system can be established to overdetermine the unknowns with the necessary stereochemical restraints (Ren et al., 2013). The analytical methods used in this work to achieve such overdetermination have been incrementally developed in the past years and recently applied to another joint analysis of the datasets of carbonmonoxy myoglobin (Ren, 2019). Time-resolved datasets collected with ultrashort pulses from an X-ray free electron laser were successfully analyzed by these methods to visualize electron density components that reveal transient heating, 3*d* electrons of the heme iron, and global vibrational motions. This analytical strategy is recapped below.

The methodological advance in this work is the refinement of each pure intermediate structure that has been deconvoluted from multiple mixtures. Structure factor amplitudes of a single conformation free of heterogeneity are overdetermined.

Given the deconvoluted structure factor amplitude set of a pure state, the standard structural refinement software with the built-in stereochemical constraints is taken full advantage of, e.g. PHENIX (Adams et al., 2010; Liebschner et al., 2019). In case that the computed deconvolution has not achieved a single pure structural species, the structural refinement is expected to make such indication.

Difference Fourier maps

A difference Fourier map is synthesized from a Fourier coefficient set of F_{light}-$F_{reference}$ with the best available phase set, often from the ground state structure. Before Fourier synthesis, F_{light} and $F_{reference}$ must be properly scaled to the same level so that the distribution of difference values is centered at zero and not skewed either way. A weighting scheme proven effective assumes that a greater amplitude of a difference Fourier coefficient F_{light}-$F_{reference}$ is more likely caused by noise than by signal (Ren et al., 2001, 2013; Šrajer et al., 2001; Ursby and Bourgeois, 1997). Both the dark and light datasets can serve as a reference in difference maps. If a light dataset at a certain delay is chosen as a reference, the difference map shows the changes since that delay time but not the changes prior to that delay. However, both the dark and light datasets must be collected in the same experiment. A cross reference from a different experimental setting usually causes large systematic errors in the difference map that would swamp the desired signals. Each difference map is masked 3.5 Å around the entire molecule of bacteriorhodopsin (bR). No lipid density is analyzed.

Meta-analysis of protein structures

Structural meta-analysis based on singular value decomposition (SVD) has been conducted in two forms. In one of them, an interatomic distance matrix is calculated from each protein structure in a related collection. SVD of a data matrix consists of these distance matrices enables a large-scale joint structural comparison but requires no structural alignment (Ren, 2013a, 2013b, 2016). In the second form, SVD is performed on a data matrix of electron densities of related protein structures (Ren, 2019; Ren et al., 2013; Schmidt et al., 2003, 2010). Both difference electron density maps that require a reference dataset from an isomorphous crystal form and simulated annealing omit maps that do not require the same unit cell and space group of the crystals are possible choices in a structural meta-analysis (Ren, 2019; Ren et al., 2013). The interatomic distances or the electron densities that SVD is performed on are called core data. Each

distance matrix or electron density map is associated with some metadata that describe the experimental conditions under which the core data are obtained, such as temperature, pH, light illumination, time delay, mutation, etc. These metadata do not enter the SVD procedure. However, they play important role in the subsequent interpretation of the SVD result. This computational method of structural analysis takes advantage of a mathematical, yet practical, definition of conformational space with limited dimensionality (Ren, 2013a). Each experimentally determined structure is a snapshot of the protein structure. A large number of such snapshots taken under a variety of experimental conditions, the metadata, would collectively provide a survey of the accessible conformational space of the protein structure and reveal its rection trajectory. Such joint analytical strategy would not be effective in early years when far fewer protein structures were determined to atomic resolution. Recent rapid growth in protein crystallography, such as in structural genomics (Berman et al., 2012; Bonvin, 2021; Chandonia and Brenner, 2006) and in serial crystallography (Glynn and Rodriguez, 2019; Schaffer et al., 2021), has supplied the necessarily wide sampling of protein structures for a joint analytical strategy to come of age. The vacancies or gaps in a conformational space between well-populated conformational clusters often correspond to less stable transient states whose conformations are difficult to capture, if not impossible. These conformations are often key to mechanistic understanding and could be explored by a back calculation based on molecular distance geometry (Ren, 2013a, 2016), the chief computational algorithm in nucleic magnetic resonance spectroscopy (NMR), and by a structure refinement based on reconstituted dataset, a major methodological advance in this work (see below). These structures refined to atomic resolution against reconstituted datasets may reveal short-lived intermediate conformation hard to be captured experimentally. Unfortunately, a protein structure refined against a reconstituted dataset currently cannot be recognized by the Protein Data Bank (PDB). Because crystallographic refinement of a macromolecular structure is narrowly defined as a correspondence from one dataset to one structure. A never-observed dataset reconstituted from a collection of experimental datasets does not match the well-established crystallographic template of PDB; let alone a refinement of crystal structure with the NMR algorithm.

A distance matrix contains M pairwise interatomic distances of a structure in the form of Cartesian coordinates of all observed atoms. An everyday example of distance

matrix is an intercity mileage chart appended to the road atlas. Differences in the molecular orientation, choice of origin, and crystal lattice among all experimentally determined structures have no contribution to the distance matrices. Due to its symmetry, only the lower triangle is necessary. A far more intimate examination of protein structures in PDB is a direct analysis of their electron density maps instead of the atomic coordinates. *M* such (difference) electron densities, often called voxels in computer graphics, are selected by a mask of interest. In the case of difference maps, only the best refined protein structure in the entire collection supplies a phase set for Fourier synthesis of electron density maps. This best structure is often the ground state structure determined by static crystallography. Other refined atomic coordinates from the PDB entries are not considered in the meta-analysis. That is to say, a meta-analysis of difference electron density maps starts from the X-ray diffraction data archived in PDB rather than the atomic coordinates interpreted from the diffraction data, which removes any potential model bias.

Singular value decomposition of (difference) electron density maps

An electron density map, particularly a difference map as emphasized here, consists of density values on an array of grid points within a mask of interest. All *M* grid points in a three-dimensional map can be serialized into a one-dimensional sequence of density values according to a specific protocol. It is not important what the protocol is as long as a consistent protocol is used to serialize all maps of the same grid setting and size, and a reverse protocol is available to erect a three-dimensional map from a sequence of *M* densities. Therefore, a set of *N* serialized maps, also known as vectors in linear algebra, can fill the columns of a data matrix **A** with no specific order, so that the width of **A** is *N* columns, and the length is *M* rows. Often, $M >> N$, thus **A** is an elongated matrix. If a consistent protocol of serialization is used, the corresponding voxel in all *N* maps occupies a single row of matrix **A**. This strict correspondence in a row of matrix **A** is important. Changes of the density values in a row from one structure to another are due to either signals, systematic errors, or noises. Although the order of columns in matrix **A** is unimportant, needless to say, the metadata associated with each column must remain in good bookkeeping.

SVD of the data matrix **A** results in $\mathbf{A} = \mathbf{UWV}^\mathrm{T}$, also known as matrix factorization. Matrix **U** has the same shape as **A**, that is, *N* columns and *M* rows. The *N* columns

contain decomposed basis components $\boldsymbol{U}_k$, known as left singular vectors of M items, where $k = 1, 2, \ldots, N$. Therefore, each component $\boldsymbol{U}_k$ can be erected using the reverse protocol to form a three-dimensional map. This decomposed elemental map can be presented in the same way as the original maps, for example, rendered in molecular graphics software such as Coot and PyMol. It is worth noting that these decomposed elemental maps or map components $\boldsymbol{U}_k$ are independent of any metadata. That is to say, these components remain constant when the metadata vary. Since each left singular vector $\boldsymbol{U}_k$ has a unit length due to the orthonormal property of SVD (see below), that is, $|\boldsymbol{U}_k| = 1$, the root mean squares (rms) of the items in a left singular vector is $1/\sqrt{M}$ that measures the quadratic mean of the items.

The second matrix **W** is a square matrix that contains all zeros except for N positive values on its major diagonal, known as singular values w_k. The magnitude of w_k is considered as a weight or significance of its corresponding component $\boldsymbol{U}_k$. The third matrix **V** is also a square matrix of $N \times N$. Each column of **V** or row of its transpose $\mathbf{V}^\mathrm{T}$, known as a right singular vector $\boldsymbol{V}_k$, contains the relative compositions of $\boldsymbol{U}_k$ in each of the N original maps. Therefore, each right singular vector $\boldsymbol{V}_k$ can be considered as a function of the metadata. Right singular vectors also have the same unit length, that is, $|\boldsymbol{V}_k| = 1$. Effectively, SVD separates the constant components independent of the metadata from the compositions that depend on the metadata.

A singular triplet denotes 1) a decomposed component $\boldsymbol{U}_k$, 2) its singular value w_k, and 3) the composition function $\boldsymbol{V}_k$. Singular triplets are often sorted in a descending order of their singular values w_k. Only a small number of n significant singular triplets identified by the greatest singular values w_1 through w_n can be used in a linear combination to reconstitute a set of composite maps that closely resemble the original ones in matrix **A**, where $n < N$. For example, the original map in the ith column of matrix **A** under a certain experimental condition can be closely represented by the ith composite map $w_1v_{1i}\boldsymbol{U}_1 + w_2v_{2i}\boldsymbol{U}_2 + \ldots + w_nv_{ni}\boldsymbol{U}_n$, where $(v_{1i}, v_{2i}, \ldots)$ is from the ith row of matrix **V**. The coefficient set for the linear combination is redefined here as $c_{ki} = w_kv_{ki}/\sqrt{M}$. The rms of the values in a map component, or the average magnitude measured by the quadratic mean, acts as a constant scale factor that resets the modified coefficients c_{ki} back to the original scale of the core data, such as Å for distance matrices and $e^-/Å^3$ for electron density maps if these units are used in the original matrix **A**.

Practically, an electron density value usually carries an arbitrary unit without a calibration, which makes this scale factor unnecessary. In the linear combination $c_{1i}\boldsymbol{U}_1 + c_{2i}\boldsymbol{U}_2 + \ldots + c_{ni}\boldsymbol{U}_n$, each component $\boldsymbol{U}_k$ is independent of the metadata while how much of each component is required for the approximation, that is, c_{ki}, depends on the metadata.

Excluding the components after $\boldsymbol{U}_n$ in this approximation is based on an assumption that the singular values after w_n are very small relative to those from w_1 through w_n. As a result, the structural information evenly distributed in all N original maps is effectively concentrated into a far fewer number of n significant components, known as information concentration or dimension reduction. On the other hand, the trailing components in matrix **U** contain inconsistent fluctuations and random noises. Excluding these components effectively rejects noises (Schmidt et al., 2003). The least-squares property of SVD guarantees that the rejected trailing components sums up to the least squares of the discrepancies between the original core data and the approximation using the accepted components.

However, no clear boundary is guaranteed between signals, systematic errors, and noises. Systematic errors could be more significant than the desired signals. Therefore, excluding some components from 1 through n is also possible. If systematic errors are correctly identified, the reconstituted map without these significant components would no longer carry the systematic errors.

The orthonormal property of SVD

The solution set of SVD must guarantee that the columns in **U** and **V**, the left and right singular vectors $\boldsymbol{U}_k$ and $\boldsymbol{V}_k$, are orthonormal, that is, $\boldsymbol{U}_h \bullet \boldsymbol{U}_k = \boldsymbol{V}_h \bullet \boldsymbol{V}_k = 0$ (ortho) and $\boldsymbol{U}_k \bullet \boldsymbol{U}_k = \boldsymbol{V}_k \bullet \boldsymbol{V}_k = 1$ (normal), where $h \neq k$ but both are from 1 to N. The orthonormal property also holds for the row vectors. As a result, each component $\boldsymbol{U}_k$ is independent of the other components. In other words, a component cannot be represented by a linear combination of any other components. However, two physical or chemical parameters in the metadata, such as temperature and pH, may cause different changes to a structure. These changes are not necessarily orthogonal. They could exhibit some correlation. Therefore, the decomposed components $\boldsymbol{U}_k$ not necessarily represent any physically or chemically meaningful changes (see below).

Due to the orthonormal property of SVD, an N-dimensional Euclidean space is established, and the first n dimensions define its most significant subspace. Each coefficient set $\boldsymbol{c}_i = (c_{1i}, c_{2i}, \ldots, c_{ni})$ of the ith composite map is located in this n-dimensional subspace. All coefficient sets for $i = 1, 2, \ldots, N$ in different linear combinations to approximate the N original maps in a least-squares sense can be represented by N points or vectors $\boldsymbol{c}_1, \boldsymbol{c}_2, \ldots, \boldsymbol{c}_N$ in the Euclidean subspace. This n-dimensional subspace is essentially the conformational space as surveyed by the jointly analyzed core data. The conformational space is presented as scatter plots with each captured structure represented as a dot located at a position determined by the coefficient set $\boldsymbol{c}_i$ of the ith observed map. When the subspace has greater dimensionality than two, multiple two-dimensional orthographical projections of the subspace are presented, such as Fig. 1a. These scatter plots are highly informative to reveal the relationship between the (difference) electron density maps and their metadata.

If two coefficient sets $\boldsymbol{c}_i \approx \boldsymbol{c}_j$, they are located close to each other in the conformational space. Therefore, these two structures i and j share two similar conformations. Two structures located far apart from each other in the conformational space are dissimilar in their conformations, and distinct in the compositions of the map components. A reaction trajectory emerges in this conformational space if the temporal order of the core data is experimentally determined (Fig. 1a). Otherwise, an order could be assigned to these structures based on an assumed smoothness of conformational changes along a reaction trajectory (Ren, 2013a, 2013b, 2016). Causation and consequence of structural motions could be revealed from the order of the structures in a series, which may further lead to structural mechanism. In addition, an off-trajectory location in the conformational space or a location between two clusters of observed structures represents a structure in a unique conformation that has never been experimentally captured. Such a hypothetical structure can be refined against a reconstituted distance matrix using molecular distance geometry (Ren, 2013a, 2013b, 2016) or a reconstituted electron density map with the method proposed below.

Rotation in SVD space

Dimension reduction is indeed effective in meta-analysis of protein structures when many datasets are evaluated at the same time. However, the default solution set of SVD carries complicated physical and chemical meanings that are not immediately obvious.

The interpretation of a basis component $\boldsymbol{U}_k$, that is, "what-does-it-mean", requires a clear demonstration of the relationship between the core data and their metadata. The outcome of SVD does not guarantee any physical meaning in a basis component. Therefore, SVD alone provides no direct answer to "what-does-it-mean", thus its usefulness is very limited to merely a mathematical construction. However, the factorized set of matrices **U**, **W**, and **V** from SVD is not a unique solution. That is to say, they are not the only solution to factorize matrix **A**. Therefore, it is very important to find one or more alternative solution sets that are physically meaningful to elucidate a structural interpretation. The concept of a rotation after SVD was introduced by Henry & Hofrichter (Henry and Hofrichter, 1992). But they suggested a protocol that fails to preserve the orthonormal and least-squares properties of SVD. The rotation protocol suggested by Ren incorporates the metadata into the analysis and combines with SVD of the core data. This rotation achieves a numerical deconvolution of multiple physical and chemical factors after a pure mathematical decomposition, and therefore, provides a route to answer the question of "what-does-it-mean" (Ren, 2019). This rotation shall not be confused with a rotation in the three-dimensional real space, in which a molecular structure resides.

A rotation in the n-dimensional Euclidean subspace is necessary to change the perspective before a clear relationship emerges to elucidate scientific findings. It is shown below that two linear combinations are identical before and after a rotation applied to both the basis components and their coefficients in a two-dimensional subspace of h and k. That is,

$$c_h\boldsymbol{U}_h + c_k\boldsymbol{U}_k = f_h\boldsymbol{R}_h + f_k\boldsymbol{R}_k, \tag{1}$$

where c_h and c_k are the coefficients of the basis components $\boldsymbol{U}_h$ and $\boldsymbol{U}_k$ before the rotation; and f_h and f_k are the coefficients of the rotated basis components $\boldsymbol{R}_h$ and $\boldsymbol{R}_k$, respectively. The same Givens rotation of an angle θ is applied to both the components and their coefficients:

$$\begin{cases} \boldsymbol{R}_h = \boldsymbol{U}_h\cos\theta - \boldsymbol{U}_k\sin\theta; \\ \boldsymbol{R}_k = \boldsymbol{U}_h\sin\theta + \boldsymbol{U}_k\cos\theta. \end{cases} \tag{2}$$

Obviously, the rotated components $\boldsymbol{R}_h$ and $\boldsymbol{R}_k$ remain mutually orthonormal and orthonormal to other components. And

$$\begin{cases} f_h = s_h t_h = c_h \cos\theta - c_k \sin\theta; \\ f_k = s_k t_k = c_h \sin\theta + c_k \cos\theta. \end{cases} \quad (3)$$

Here $s_{h|k} = \sqrt{\sum f_{h|k}^2}$ are the singular values that replace w_h and w_k, respectively, after the rotation. They may increase or decrease compared to the original singular values so that the descending order of the singular values no longer holds. $\boldsymbol{T}_{h|k} = (t_{h|k1}, t_{h|k2}, \ldots, t_{h|kN}) = (f_{h|k1}, f_{h|k2}, \ldots, f_{h|kN})/s_{h|k}$ are the right singular vectors that replace $\boldsymbol{V}_h$ and $\boldsymbol{V}_k$, respectively. $\boldsymbol{T}_h$ and $\boldsymbol{T}_k$ remain mutually orthonormal after the rotation and orthonormal to other right singular vectors that are not involved in the rotation.

Eq. 1 holds because the dot product of two vectors does not change after both vectors rotate the same angle. To prove Eq. 1 in more detail, Eqs. 2 and 3 are combined and expanded. All cross terms of sine and cosine are self-canceled:

$$\begin{aligned} f_h\boldsymbol{R}_h + f_k\boldsymbol{R}_k &= (c_h\cos\theta - c_k\sin\theta)(\boldsymbol{U}_h\cos\theta - \boldsymbol{U}_k\sin\theta) + (c_h\sin\theta + c_k\cos\theta)(\boldsymbol{U}_h\sin\theta + \boldsymbol{U}_k\cos\theta) \\ &= c_h\boldsymbol{U}_h\cos^2\theta + c_k\boldsymbol{U}_k\sin^2\theta + c_h\boldsymbol{U}_h\sin^2\theta + c_k\boldsymbol{U}_k\cos^2\theta \pm c_h\boldsymbol{U}_k\sin\theta\cos\theta \pm c_k\boldsymbol{U}_h\sin\theta\cos\theta \\ &= c_h\boldsymbol{U}_h(\cos^2\theta + \sin^2\theta) + c_k\boldsymbol{U}_k(\sin^2\theta + \cos^2\theta) \\ &= c_h\boldsymbol{U}_h + c_k\boldsymbol{U}_k \end{aligned}$$

A rotation in two-dimensional subspace of *h* and *k* has no effect in other dimensions, as the orthonormal property of SVD guarantees. Multiple steps of rotations can be carried out in many two-dimensional subspaces consecutively to achieve a multi-dimensional rotation. A new solution set derived from a rotation retains the orthonormal property of SVD. The rotation in the Euclidean subspace established by SVD does not change the comparison among the core data of protein structures. Rather it converts one solution set $\mathbf{A} = \mathbf{UWV}^\mathrm{T}$ to other alternative solutions $\mathbf{A} = \mathbf{RST}^\mathrm{T}$ so that an appropriate perspective can be found to elucidate the relationship between the core data and metadata clearly and concisely.

For example, if one physical parameter could be reoriented along a single dimension *k* but not involving other dimensions by a rotation, it can be convincingly shown that

the left singular vector $\boldsymbol{U}_k$ of this dimension illustrates the structural impact by this physical parameter. Before this rotation, the same physical parameter may appear to cause structural variations along several dimensions, which leads to a difficult interpretation. Would a proper rotation establish a one-on-one correspondence from all physical or chemical parameters to all the dimensions? It depends on whether each parameter induces an orthogonal structural change, that is, whether structural responses to different parameters are independent or correlated among one another. If structural changes are indeed orthogonal, it should be possible to find a proper rotation to cleanly separate them in different dimensions. Otherwise, two different rotations are necessary to isolate two correlated responses, but one at a time.

For another example, if the observed core datasets form two clusters in the conformational space, a rotation would be desirable to separate these clusters along a single dimension *k* but to align these clusters along other dimensions. Therefore, the component $\boldsymbol{U}_k$ is clearly due to the structural transition from one cluster to the other. Without a proper rotation, the difference between these clusters could be complicated with multiple dimensions involved. A deterministic solution depends on whether a clear correlation exists between the core data and metadata. A proper rotation may require a user decision. A wrong choice of rotation may select a viewpoint that hinders a concise conclusion. However, it would not alter the shape of the reaction trajectory, nor create or eliminate an intrinsic structural feature. A wrong choice of rotation cannot eliminate the fact that a large gap exists between two clusters of observed core datasets except that these clusters are not obvious from that viewpoint. A different rotation may reorient the perspective along another direction. But the structural conclusion would be equivalent. See example of before and after a rotation in (Ren, 2016).

This rotation procedure finally connects the core crystallographic datasets to the metadata of experimental conditions and accomplishes the deconvolution of physical or chemical factors that are not always orthogonal to one another after a mathematical decomposition. SVD analysis presented in this paper employs rotations extensively except that no distinction is made in the symbols of components and coefficients before and after a rotation except in this section. This method is widely applicable in large-scale structural comparisons. Furthermore, Ren rotation after SVD is not limited to

crystallography and may impact other fields wherever SVD is used. For example, SVD is frequently applied to spectroscopic data, images, and genetic sequence data.

Structural refinement against reconstituted dataset

The linear combination $\Delta\rho(t) = f_1(t)\boldsymbol{R}_1 + f_2(t)\boldsymbol{R}_2 + \ldots + f_n(t)\boldsymbol{R}_n$ after a rotation reconstitutes one of the observed difference maps at a specific time point *t*. This time-dependent difference map depicts an ever-evolving mixture of many excited species. A reconstituted difference map $\Delta\rho$(E) for a time-independent, pure, excited species E = intermediate I′, I, J′, and J deconvoluted from many mixtures would take the same form except that only one or very few coefficients remain nonzero if a proper rotation has been found (Table S2). In order to take advantage of the mature refinement software for macromolecular structures with extensive stereochemical restraints, a set of structure factor amplitudes is needed. Therefore, it is necessary to reconstitute a set of structure factor amplitudes that would produce the target difference map $\Delta\rho$(E) based on a known structure at the ground state. First, an electron density map of the structure at the ground state is calculated. This calculated map is used as a base map. Second, this base map of the ground state is combined with the positive and negative densities in the target difference map $\Delta\rho$(E) so that the electron densities at the ground state are skewed toward the intermediate state. Third, structure factors are calculated from the combined map. Finally, the phase set of the calculated structure factors is discarded, and the amplitudes are used to refine a single conformation of the intermediate species E that $\Delta\rho$(E) represents.

This protocol following the SVD and Ren rotation of components achieves a refinement of a pure structural species without the need of alternative conformations. Several points are noteworthy. First, the minimization protocol in this refinement is performed against a numerically reconstituted amplitude set that has never been directly measured from a crystal. This reconstituted dataset could be considered as an extrapolated dataset "on steroids" if compared to the traditional extrapolation of small differences, such as, the Fourier coefficient set to calculate a 3Fo-2Fc map, a technique often used to overcome a partial occupancy of an intermediate structure. An extrapolation of small differences is not directly observed either but computed by an exaggeration of the observed difference based on an assumption that the intermediate state is partially occupied, such as the doubling of the observed difference in 3Fo-2Fc =

Fo + 2(Fo-Fc). In contrast to the conventional technique of extrapolation, the deconvolution method applied here is an interpolation among many experimental datasets rather than an extrapolation. Secondly, the deconvolution is a simultaneous solution of multiple intermediate states mixed together instead of solving a single excited state.

Second, a map calculated from the ground state structure is chosen as the base map instead of an experimental map such as Fo or 2Fo-Fc map. If the second step of the protocol is skipped, that is, no difference map is combined with the ground state map, the refinement would result in an *R* factor of nearly zero, since the refinement is essentially against the calculated structure factors (bR in Table S2). This is to say, the residuals of the refinement are solely due to the difference component instead of the base map. This is desirable since errors in the static structure of the ground state are gauged during its own refinement. On the other hand, if an experimental map is chosen as a base map, the refinement *R* factors would reflect errors in both the base map and the difference map, which leads to a difficulty in an objective evaluation of this refinement protocol.

Third, the combination of the base map and a difference map is intended to represent a pure intermediate species. Therefore, alternative conformations in structural refinement that model a mixture of species would defeat this purpose. However, this combined map could be very noisy and may not represent a single species without a proper rotation. This is particular the case, if the target difference map $\Delta\rho$ is not derived from an SVD analysis and Ren rotation. The SVD analysis identifies many density components that are inconsistent among all observed difference maps and excludes them, which greatly reduces the noise content. Therefore, this refinement protocol may not be very successful without an SVD analysis. Another source of noise originates from the phase set of the structure factors. Prior to the refinement of the intermediate structure, the phase set remains identical to that of the ground state. This is far from the reality when an intermediate structure involves widespread changes, such as those refined in this study. If the rotation after SVD is not properly selected, the target difference map would remain as a mixture minus the ground state. Therefore, the refinement of a single conformation would encounter difficulty or significant residuals, as judged by the *R* factors, the residual map, and the

refined structure. A proper solution to this problem is a better SVD solution by Ren rotation rather than alternative conformations. A successful refinement of near perfect *trans* or *cis* double bonds is a good sign to indicate that the reconstituted amplitude set after a rotation reflects a relatively homogeneous structure. If a double bond could not be refined well to near perfect *trans* or *cis* configuration, the dataset of structure factor amplitudes is likely from a mixture of heterogeneous configurations, which occurred frequently in previous studies of bR and photoactive yellow protein (Jung et al., 2013; Lanyi and Schobert, 2007; Nogly et al., 2018). It has been a great difficulty in crystallographic refinement in general that a heterogeneous mixture of conformations cannot be unambiguously refined even with alternative conformations. This difficulty becomes more severe when a mixture involves more than two conformations or when some conformations are very minor.

Lastly, the refinement protocol proposed here could be carried out in the original unit cell and space group of the crystal at the ground state. However, this is not always applicable as the original goal of the meta-analysis is a joint examination of all available structures from a variety of crystal forms. It would be highly desirable to evaluate difference maps of the same or similar proteins from non-isomorphous crystals together by SVD. Alternatively, the refinement protocol could also be performed in the space group of P1 with a virtual unit cell large enough to hold the structure, which is the option in this study (Table S2). This is to say, the entire analysis of SVD-rotation-refinement presented here could be extracted and isolated from the original crystal lattices, which paves the way to future applications to structural data acquired by experimental techniques beyond crystallography, most attractively, to single particle reconstruction in cryo electron microscopy.

References

Adams, P.D., Afonine, P.V., Bunkóczi, G., Chen, V.B., Davis, I.W., Echols, N., Headd, J.J., Hung, L.-W., Kapral, G.J., Grosse-Kunstleve, R.W., et al. (2010). PHENIX: a comprehensive Python-based system for macromolecular structure solution. Acta Crystallogr. D Biol. Crystallogr. *D66*, 213–221. https://doi.org/10.1107/S0907444909052925.

Berman, H.M., Kleywegt, G.J., Nakamura, H., and Markley, J.L. (2012). The Protein Data Bank at 40: Reflecting on the past to prepare for the future. Structure *20*, 391–396. https://doi.org/10.1016/j.str.2012.01.010.

Bonvin, A.M.J.J. (2021). 50 years of PDB: a catalyst in structural biology. Nat. Methods *18*, 448–449. https://doi.org/10.1038/s41592-021-01138-y.

Chandonia, J.-M., and Brenner, S.E. (2006). The impact of structural genomics: expectations and outcomes. Science *311*, 347–351. https://doi.org/10.1126/science.1121018.

Glynn, C., and Rodriguez, J.A. (2019). Data-driven challenges and opportunities in crystallography. Emerg. Top. Life Sci. ETLS20180177. https://doi.org/10.1042/ETLS20180177.

Henry, E.R., and Hofrichter, J. (1992). Singular value decomposition: Application to analysis of experimental data. In Numerical Computer Methods, (Academic Press), pp. 129–192.

Jung, Y.O., Lee, J.H., Kim, J., Schmidt, M., Moffat, K., Šrajer, V., and Ihee, H. (2013). Volume-conserving trans–cis isomerization pathways in photoactive yellow protein visualized by picosecond X-ray crystallography. Nat. Chem. *5*, 212–220. https://doi.org/10.1038/nchem.1565.

Lanyi, J.K., and Schobert, B. (2007). Structural changes in the L photointermediate of bacteriorhodopsin. J. Mol. Biol. *365*, 1379–1392. https://doi.org/10.1016/j.jmb.2006.11.016.

Liebschner, D., Afonine, P.V., Baker, M.L., Bunkóczi, G., Chen, V.B., Croll, T.I., Hintze, B., Hung, L.-W., Jain, S., McCoy, A.J., et al. (2019). Macromolecular structure determination using X-rays, neutrons and electrons: recent developments in Phenix. Acta Crystallogr. Sect. Struct. Biol. *75*, 861–877. https://doi.org/10.1107/S2059798319011471.

Nogly, P., Weinert, T., James, D., Carbajo, S., Ozerov, D., Furrer, A., Gashi, D., Borin, V., Skopintsev, P., Jaeger, K., et al. (2018). Retinal isomerization in bacteriorhodopsin captured by a femtosecond x-ray laser. Science *361*, eaat0094. https://doi.org/10.1126/science.aat0094.

Ren, Z. (2013a). Reaction trajectory revealed by a joint analysis of Protein Data Bank. PLoS ONE *8*, e77141. https://doi.org/10.1371/journal.pone.0077141.

Ren, Z. (2013b). Reverse engineering the cooperative machinery of human hemoglobin. PLoS ONE *8*, e77363. https://doi.org/10.1371/journal.pone.0077363.

Ren, Z. (2016). Molecular events during translocation and proofreading extracted from 200 static structures of DNA polymerase. Nucleic Acids Res. *6*, 1–13. https://doi.org/10.1093/nar/gkw555.

Ren, Z. (2019). Ultrafast structural changes decomposed from serial crystallographic data. J. Phys. Chem. Lett. *10*, 7148–7163. https://doi.org/10.1021/acs.jpclett.9b02375.

Ren, Z., Perman, B., Srajer, V., Teng, T.-Y., Pradervand, C., Bourgeois, D., Schotte, F., Ursby, T., Kort, R., Wulff, M., et al. (2001). A molecular movie at 1.8 Å resolution displays the photocycle of photoactive yellow protein, a eubacterial blue-light receptor, from nanoseconds to seconds. Biochemistry *40*, 13788–13801. https://doi.org/10.1021/bi0107142.

Ren, Z., Chan, P.W.Y., Moffat, K., Pai, E.F., Royer, W.E., Šrajer, V., and Yang, X. (2013). Resolution of structural heterogeneity in dynamic crystallography. Acta Cryst *D69*, 946–959. https://doi.org/10.1107/S0907444913003454.

Schaffer, J.E., Kukshal, V., Miller, J.J., Kitainda, V., and Jez, J.M. (2021). Beyond X-rays: an overview of emerging structural biology methods. Emerg. Top. Life Sci. ETLS20200272. https://doi.org/10.1042/ETLS20200272.

Schmidt, M., Rajagopal, S., Ren, Z., and Moffat, K. (2003). Application of singular value decomposition to the analysis of time-resolved macromolecular X-ray data. Biophys. J. *84*, 2112–2129. https://doi.org/10.1016/S0006-3495(03)75018-8.

Schmidt, M., Graber, T., Henning, R., and Srajer, V. (2010). Five-dimensional crystallography. Acta Crystallogr. A *66*, 198–206. https://doi.org/10.1107/S0108767309054166.

Šrajer, V., Ren, Z., Teng, T.-Y., Schmidt, M., Ursby, T., Bourgeois, D., Pradervand, C., Schildkamp, W., Wulff, M., and Moffat, K. (2001). Protein conformational relaxation and ligand migration in myoglobin: A nanosecond to millisecond molecular movie from time-resolved Laue X-ray diffraction. Biochemistry *40*, 13802–13815. https://doi.org/10.1021/bi010715u.

Ursby, T., and Bourgeois, D. (1997). Improved estimation of structure-factor difference amplitudes from poorly accurate data. Acta Crystallogr. A *53*, 564–575. https://doi.org/10.1107/S0108767397004522.

Supplementary Tables

Table S1. Datasets analyzed in this work

Publication	PDB	Label	Resolution	Main conclusions	New findings in this work
Nogly et al. Science 361, eaat0094, 2018	6g7h<br>6g7i<br>6g7j<br>6g7k	dark6<br>49-406fs<br>457-646fs<br>10ps	1.5 Å<br>1.9 Å<br>1.9 Å<br>1.9 Å	Retinal fully isomerizes by 10 ps. But the SB water dissociates earlier.	The short-delay datasets contribute to the structures of I′ → I → J′ → J. Photoisomerization in J′; retinal binding pocket expansion before 1 ps in I and contraction at ~20 ps in J
Kovacs et al. Nat. Commun. 10, 3177, 2019	6ga1<br>6ga2<br>6rmk<br>6ga7<br>6ga8<br>6ga9<br>6gaa<br>6gab<br>6gac<br>6gad<br>6gae<br>6gaf<br>6gag<br>6gah<br>6gai<br>6ga4<br>6ga5<br>6ga6	dark1<br>dark2<br>dark3<br>240fs<br>330fs<br>390fs<br>430fs<br>460fs<br>490fs<br>530fs<br>560fs<br>590fs<br>630fs<br>680fs<br>740fs<br>1ps<br>3ps<br>10ps	1.7 Å<br>1.8 Å<br>1.8 Å<br>1.8 Å<br>1.8 Å<br>1.8 Å<br>1.8 Å<br>1.8 Å<br>1.8 Å<br>1.8 Å<br>1.8 Å<br>1.8 Å<br>1.8 Å<br>1.8 Å<br>1.8 Å<br>1.8 Å<br>1.9 Å<br>1.8 Å	The exceedingly high power density of the pump laser causes two-photon absorption. Vibrational motions were observed.	The sub-ps datasets exhibit extensive vibrations at various frequencies. The vibrational signals are widespread over the entire bR molecule and not associated with any structural elements. Therefore, it is concluded that these global vibrations are intrinsic properties of bR induced by short laser pulses. The vibrational signals are more prominent under higher power density of the laser pulses. However, these vibrations are irrelevant to the light-driven proton pumping function of bR.

Table S2. Refinement statistics						
Intermediate		bR	I′	I	J′	J
Time period		0-	< 50 fs	40-700 fs	0.5-2 ps	1-30 ps
PDB-Dev* entry		PDBDEV_00000129		138	139	140
Coefficient	c_{10}	0	0	3,300	0	-4,200
	c_{14}	0	2,000	2,700	2,700	2,000
	c_{17}	0	3,000	0	-1,300	-300
Starting model		PDB 6g7h				
Resolution range		50-2.1 Å				
Space group		P1				
Unit cell		$a = b = 62.32$ Å; $c = 111.10$ Å; $\alpha = \beta = 90°$; and $\gamma = 120°$				
Unique reflections		80,354 in working set + 4,236 in test set = 84,590 total				
Completeness		95% in working set + 5% in test set = 100% reconstituted				
R (%)		1.8	29.4	31.0	29.1	30.0
R_{free} (%)		1.9	31.1	32.4	30.4	30.7
Refined content		230 protein residues + retinal + water molecules				
Number of atoms		1,798	1,795	1,798	1,796	1,795
Water molecules		8	5	8	6	5
RMSD bonds (Å)		0.005	0.009	0.009	0.009	0.009
RMSD angles (°)		0.793	1.206	1.105	1.085	1.068
Rama. favored (%)		98.7	96.5	95.6	96.1	96.5
Rama. outliers (%)		0.0	0.0	0.4	0.4	0.4
Clash score		4	9	5	4	6

*pdb-dev.wwwpdb.org

Supplementary Figures and Legends

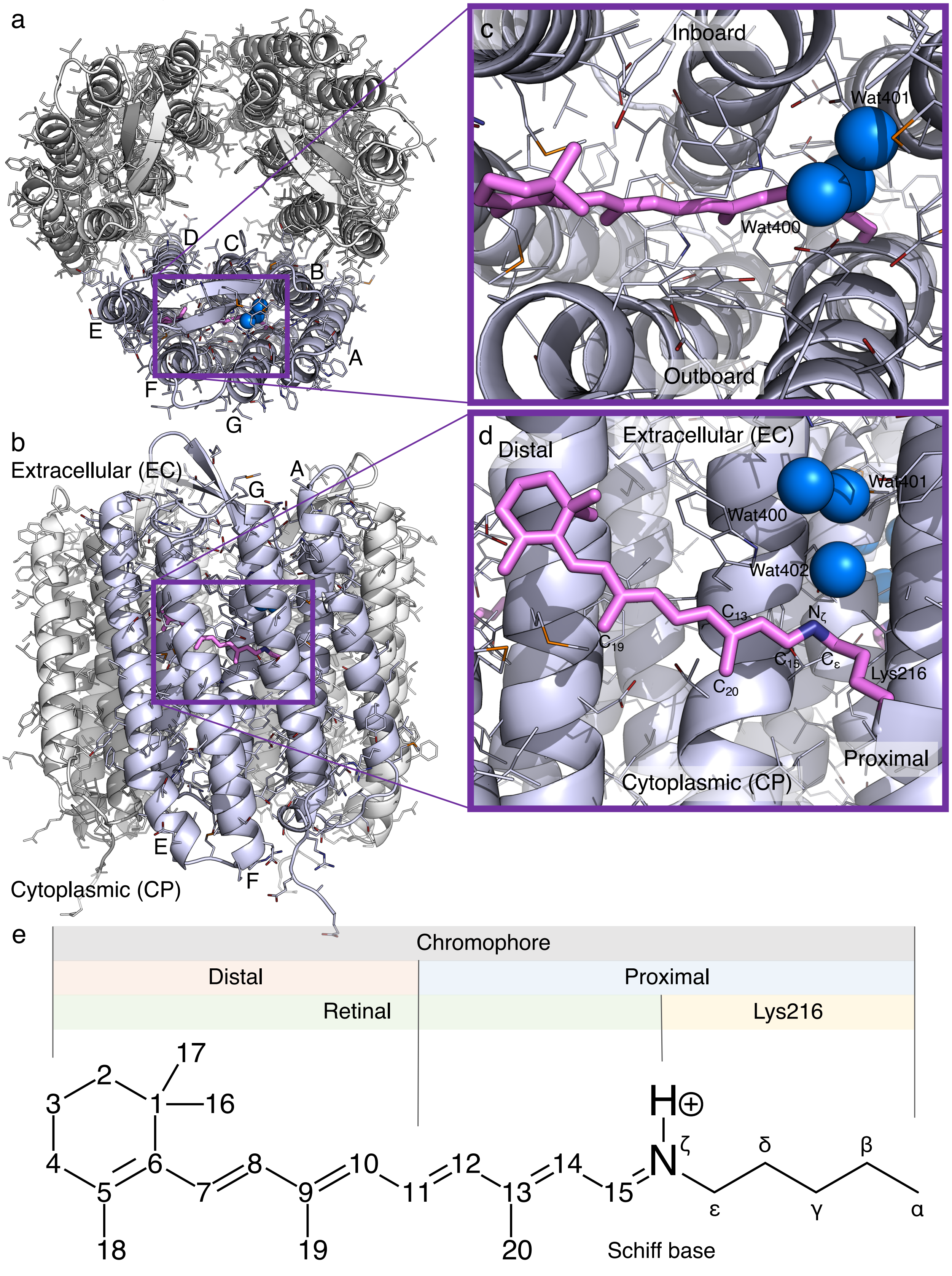

Figure S1. Orientations in bacteriorhodopsin. (a) Bacteriorhodopsin (bR) trimer viewed from the extracellular (EC) side along the three-fold axis. (b) An orthographical view to (a) looking from the outside of the trimer. (c and d) Two orthographical views of the retinal chromophore looking along the three-fold and normal to the three-fold axis. The plane of retinal is largely parallel to the three-fold axis. Therefore, two sides of the plane are called inboard and outboard with respect to the three-fold axis. The direction toward the anchor Lys216 is called proximal. The β-ionone ring direction is therefore distal. (e) Chemical structure of retinal incorporated to its anchor Lys216. The atom numbers and various segment names are marked.

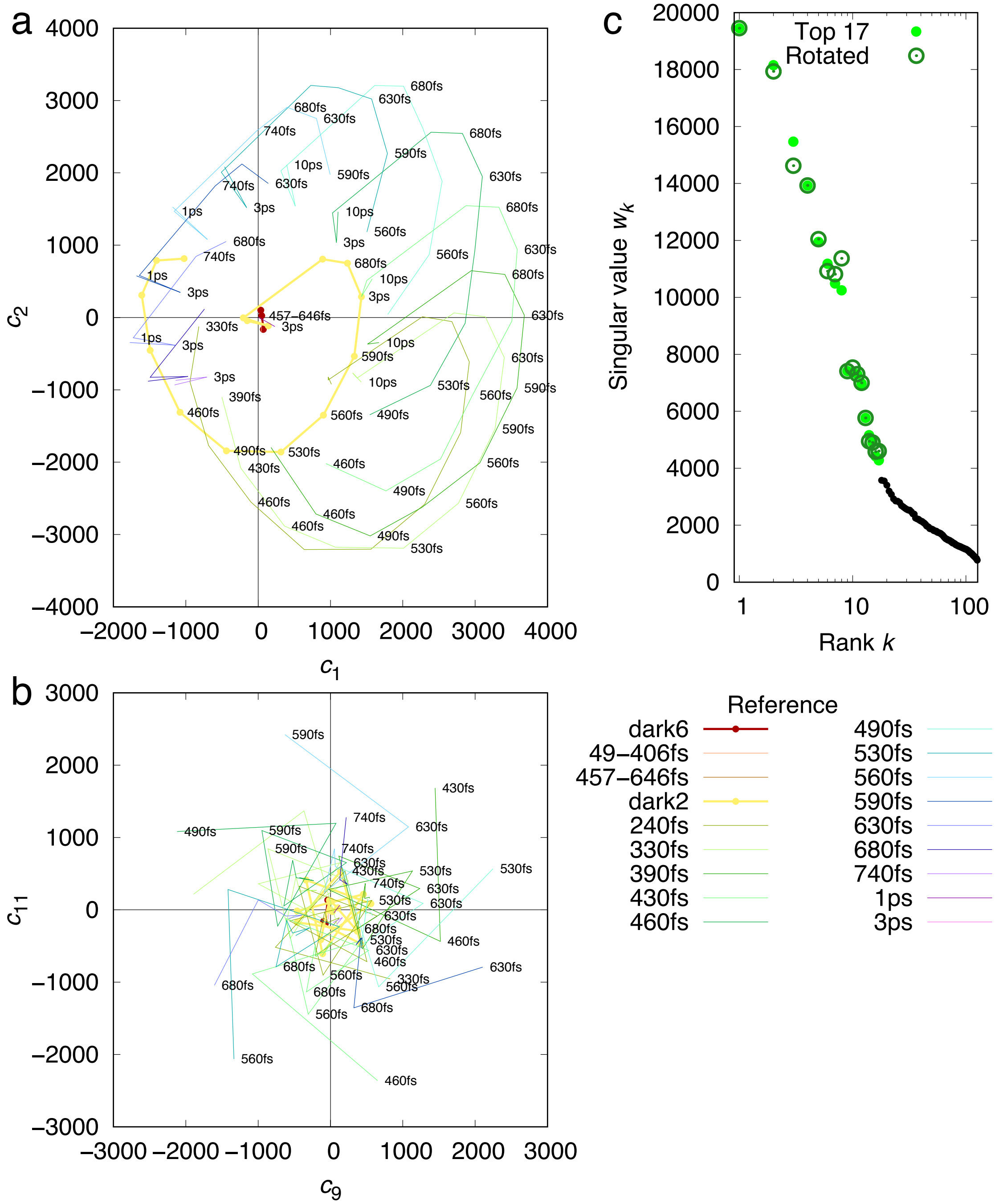

Figure S2. SVD applied to difference Fourier maps. Difference Fourier maps at the short delays $t \le 10$ ps are decomposed into component maps. Each difference map at a time delay t can be represented by a linear combination of these components, $c_1(t)\boldsymbol{U}_1 + c_2(t)\boldsymbol{U}_2 + \ldots$, where $\boldsymbol{U}_k$ are the time-independent components and $c_k(t)$ are their corresponding time-dependent coefficients (Methods). (a and b) Two example plots show circular correlations between c_1 and c_2, c_9 and c_{11}. These circular correlations

indicate two-dimensional oscillations. Each colored trace represents difference maps in a time series calculated with a common reference. Those time series with a dark reference are plotted with thick lines. Other series are in thin lines. (c) Singular values before and after Ren rotation (Ren, 2016, 2019) (Methods). Singular values derived from SVD indicate the significance of the components. 17 of them stand out.

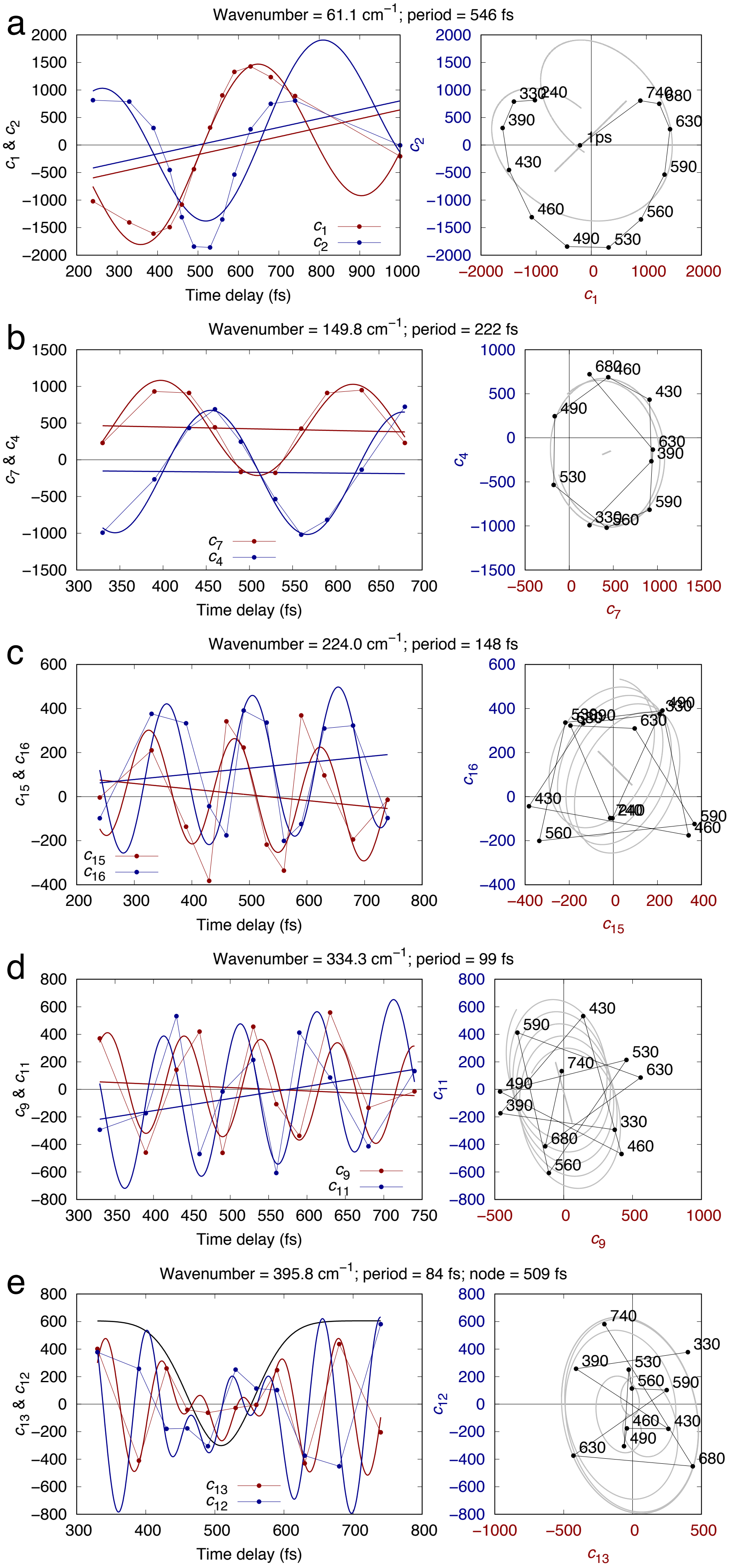
a
Wavenumber = 61.1 cm−1; period = 546 fs
c1 & c2
Time delay (fs)
c1
c2
b
Wavenumber = 149.8 cm−1; period = 222 fs
c7 & c4
Time delay (fs)
c7
c4
c
Wavenumber = 224.0 cm−1; period = 148 fs
c15 & c16
Time delay (fs)
c15
c16
d
Wavenumber = 334.3 cm−1; period = 99 fs
c9 & c11
Time delay (fs)
c9
c11
e
Wavenumber = 395.8 cm−1; period = 84 fs; node = 509 fs
c13 & c12
Time delay (fs)
c13
c12

Figure S3. Oscillations of SVD components. The coefficients of ten components c_1, c_2; c_4, c_7; c_{15}, c_{16}; c_9, c_{11}; and c_{12}, c_{13} are found oscillating at frequencies ranging from 60 to 400 cm^{-1}. Each pair of the coefficients oscillate at a common frequency. These frequencies are 61±2, 150±3, 224±7, 334±8, and 396±3 cm^{-1}, respectively. These coefficients are plotted against the time delay t (left) and against each other in a pair (right). Each coefficient is fitted with a sine function around a straight baseline $c_k = a \sin\left(\frac{2\pi t}{T} + \varphi\right) + b + ct$. Both the fitted function and the baseline are plotted. The amplitude a for the last pair of coefficients c_{12} and c_{13} are replaced with a Gaussian function $a - \exp\left(-\frac{(t-t_0)^2}{\tau^2}\right)$ to implement a node at t_0 = 509±5 fs (e).

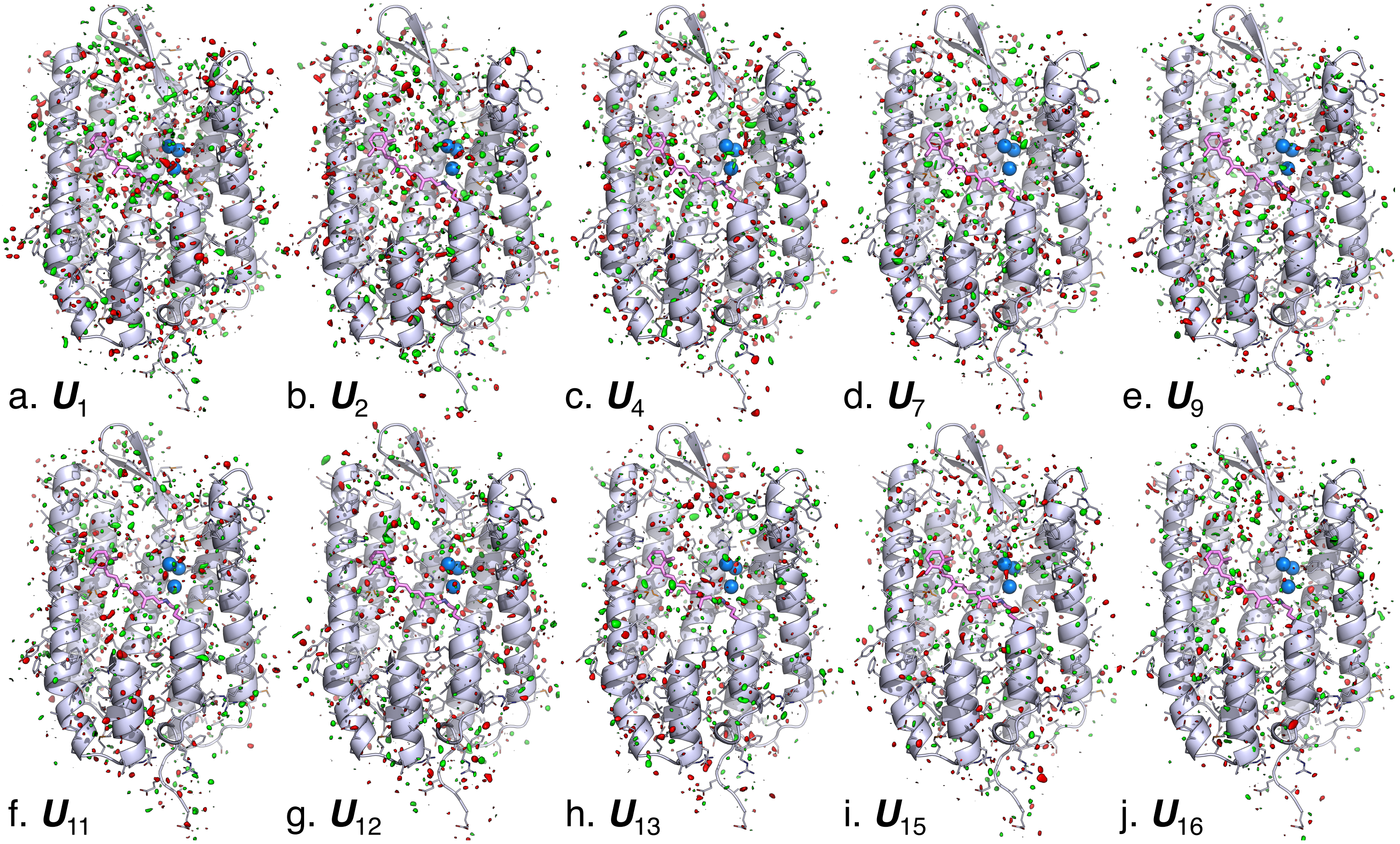

Figure S4. No structural signal in oscillating components. Ten oscillating components are contoured at $\pm 3\sigma$ in green and red, respectively. The main chain and side chains of the protein are rendered with ribbon and sticks, respectively. The retinal and Lys216 are in purple sticks. Several key waters are in blue spheres. Parts of the structure are omitted to reveal more of the interior. Despite that the time-dependent coefficients to these components contain strong oscillatory signals (Figs. S2 and S3), these components themselves display no obvious association with any structural features such as the retinal or secondary structures. They are in stark contrast to the signal distributions of the non-oscillating components (Figs. 2ab, 3c, and S5).

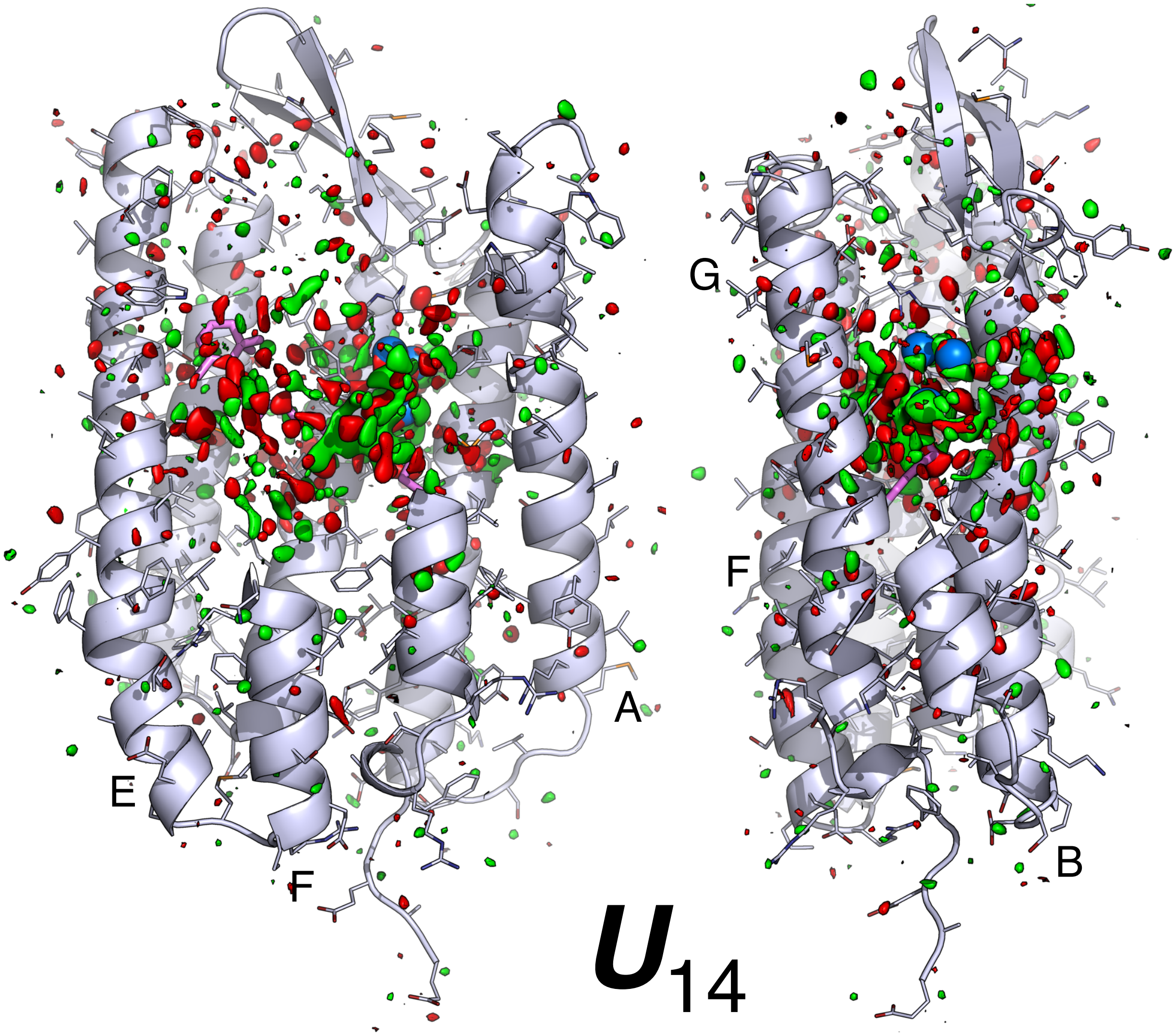

Figure S5. Two orthographical views of component map $\boldsymbol{U}_{14}$. The main chain and side chains of the protein are rendered with ribbons and sticks, respectively. The retinal and Lys216 are in purple sticks. Several key waters are in blue spheres. Parts of the structure are omitted to reveal more of the interior. The map is contoured at $\pm 3\sigma$ in green and red, respectively. The signals are largely associated with the chromophore and its immediate vicinity.

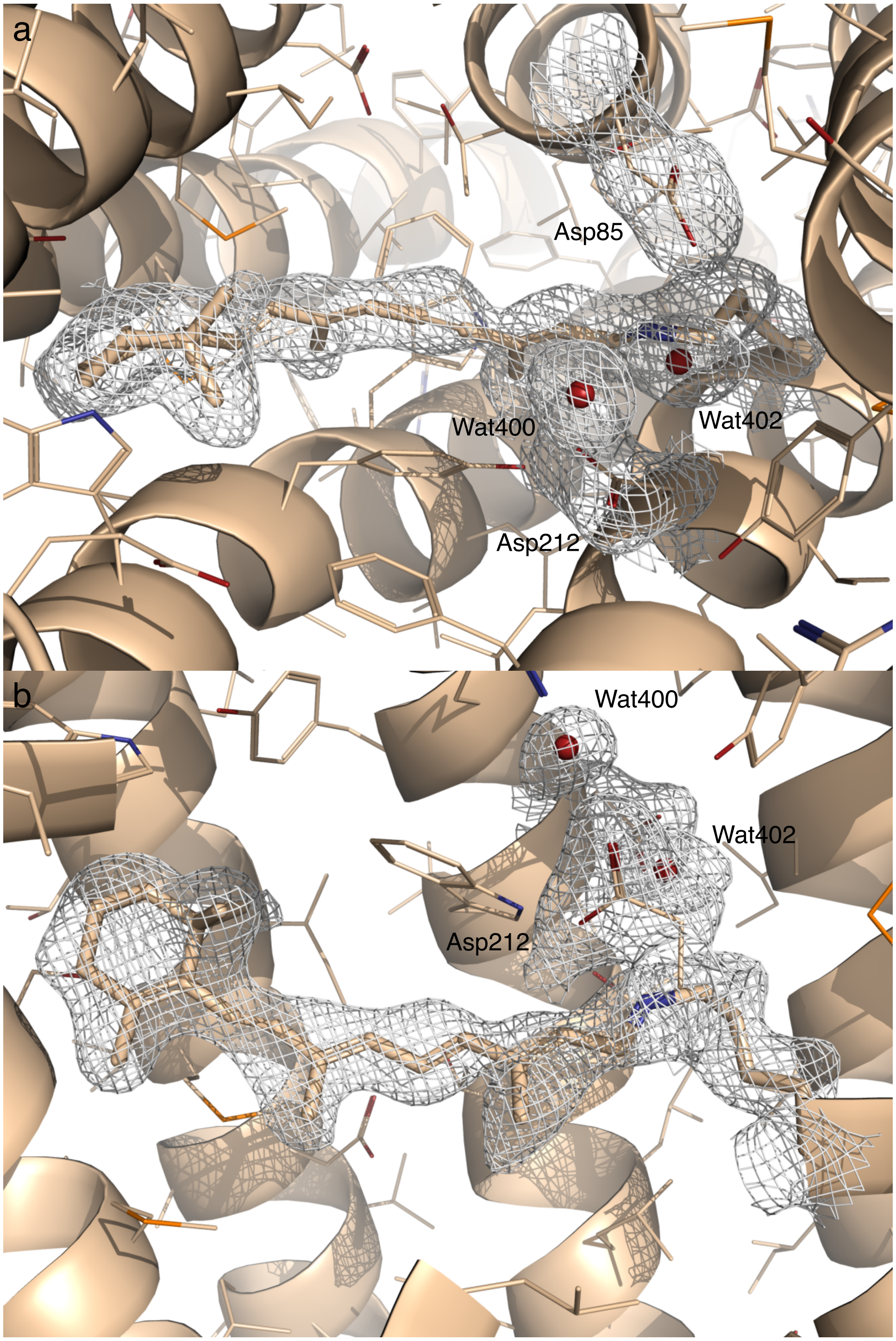

Figure S6. Two orthographical views of the 2Fo-Fc map of I′ contoured at 3.5σ. Here Fo is the reconstituted structure factor amplitudes rather than observed amplitudes (Table S2). Fc is the structure factor amplitudes calculated from the refine structure (Methods).

The same protocol applies to the Fourier synthesis of 2Fo-Fc maps of other intermediates (Figs. S7, S11, and S12).

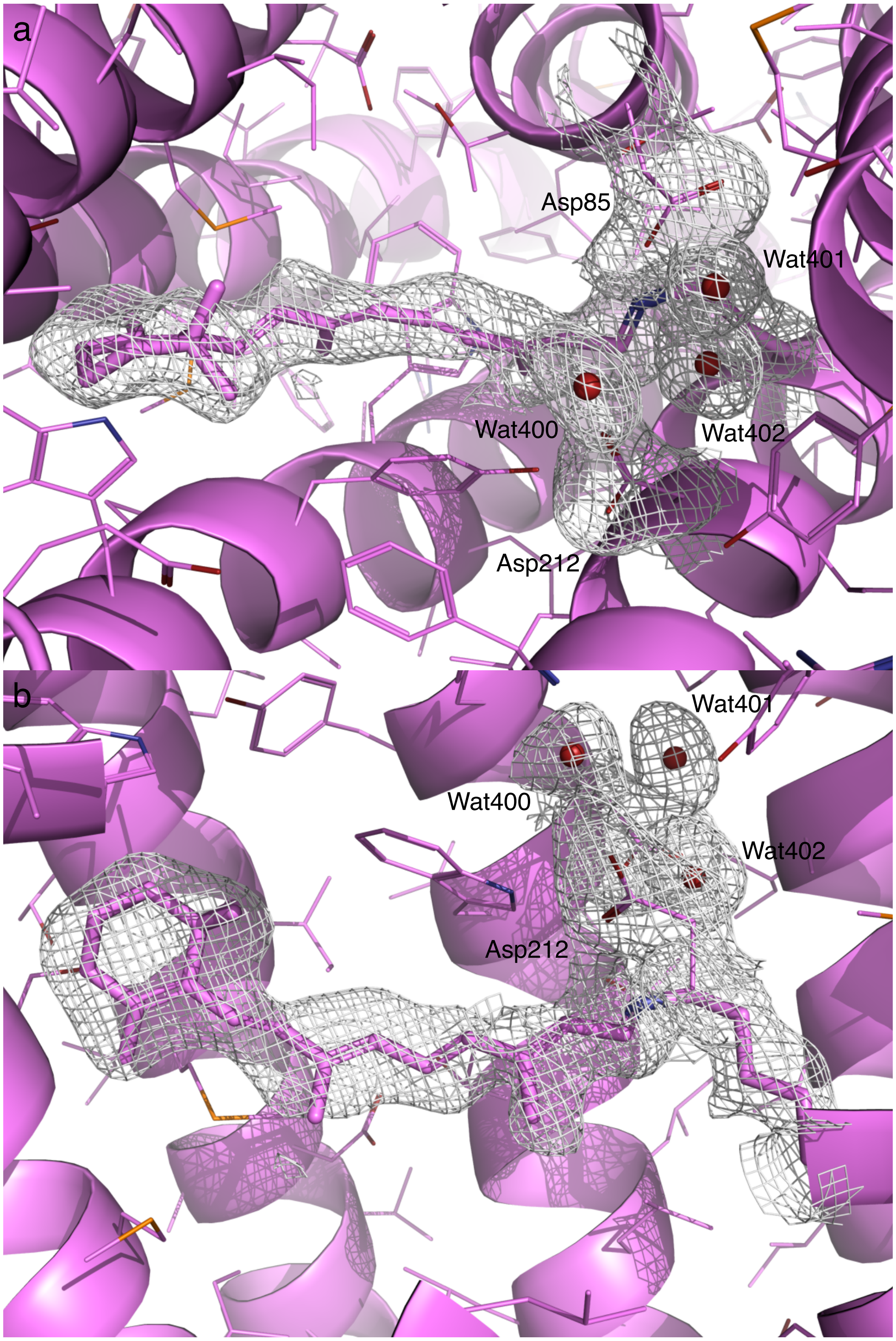

Figure S7. Two orthographical views of the 2Fo-Fc map of I contoured at 3σ. Here Fo is the reconstituted structure factor amplitudes rather than observed amplitudes (Table S2). Fc is the structure factor amplitudes calculated from the refine structure (Methods).

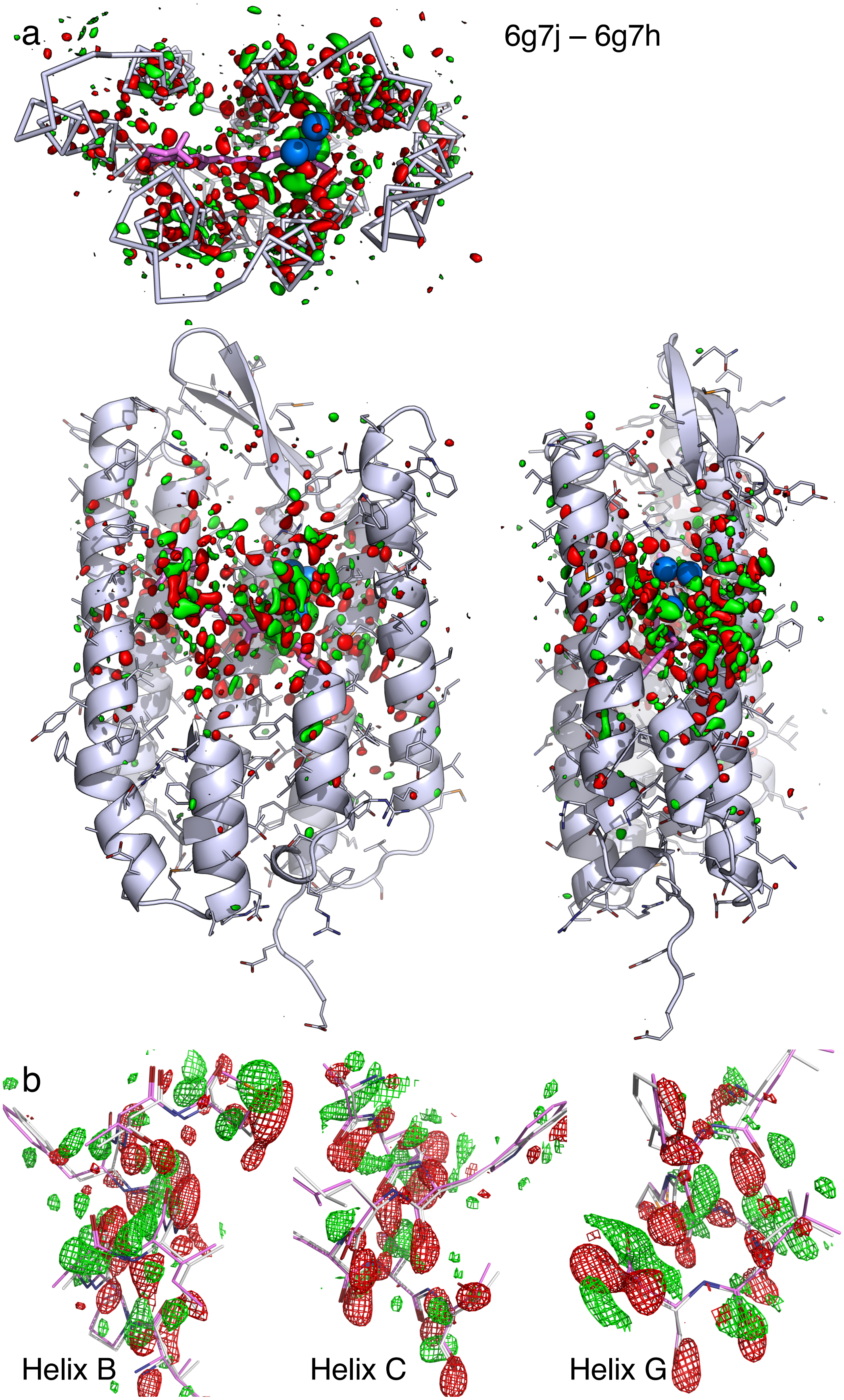
a
6g7j – 6g7h
b
Helix B
Helix C
Helix G

Figure S8. Raw difference Fourier map at 457-646 fs. This difference Fourier map is calculated from the dataset 6g7j at the time point of 457-646 fs by subtracting the dark dataset 6g7h. The map is contoured at $\pm 3\sigma$ in green and red, respectively. This map is prior to SVD analysis. Compared with $\boldsymbol{U}_{10}$ (Fig. 3c) and the reconstituted map (Fig. 3d), it is clear that this is the original source of the widespread signals except that the σ value of this map is higher than those after SVD. (a) The raw difference map contoured in the entire molecule shows the association of the signals with the structural elements at an excellent signal-to-noise ratio. (b) Details of the raw difference map show displacements of helices. The raw difference map is largely the same as the reconstituted map (Fig. 3d).

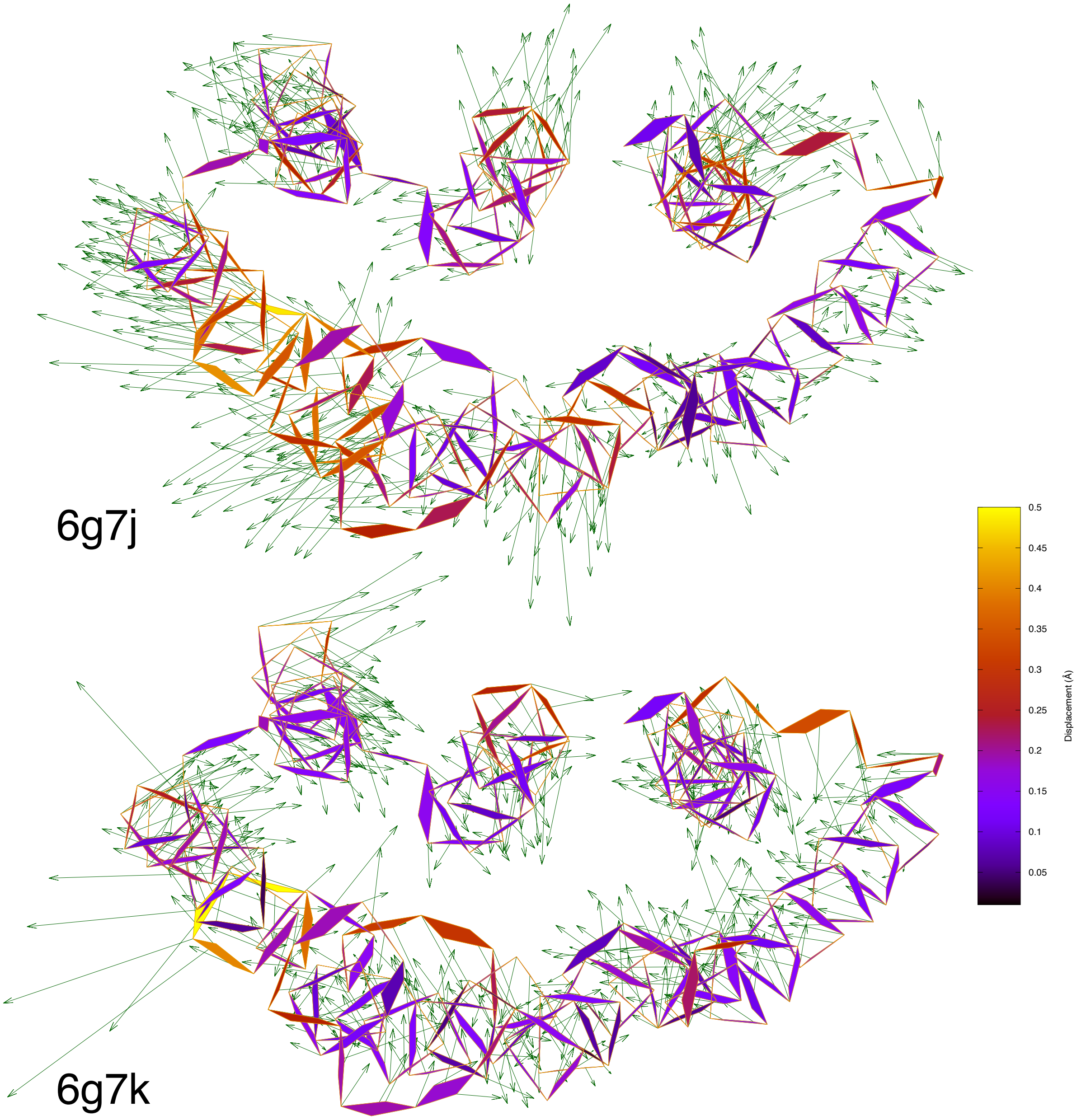

Figure S9. Refined structures at 457-646 fs and 10 ps compared to the ground state. The refined structures of 6g7j and 6g7k and compared with the resting state 6g7h viewed along the trimer three-fold axis from the extracellular side. Atomic displacements in the main chain from bR to 6g7j and 6g7k are color coded and marked by arrows with lengths 20× of the actual displacements. Compared to the structures of I and J species (Fig. 3e), smaller expansion and slight contraction were also captured.

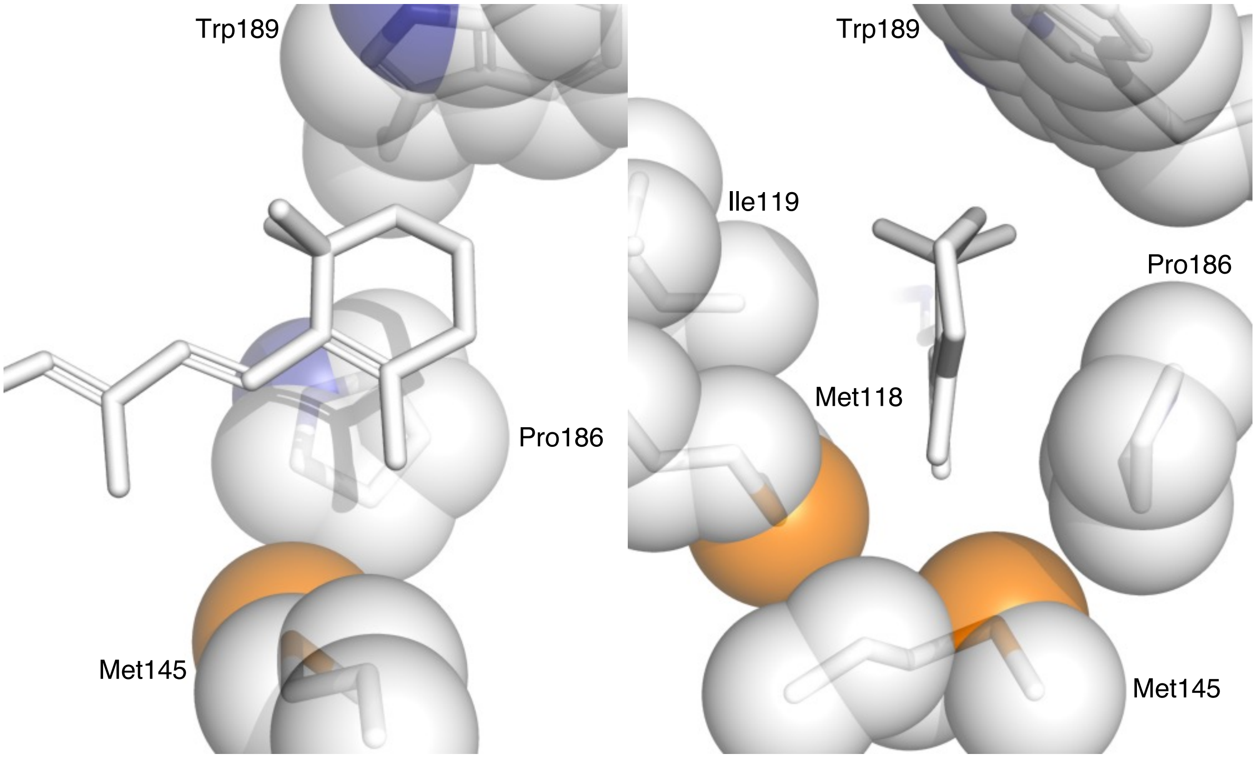

Figure S10. Retinal binding pocket at the distal end. Two orthographical views of the retinal at its distal end. The closest contacts are 3.7 Å to Ile119 and Pro186.

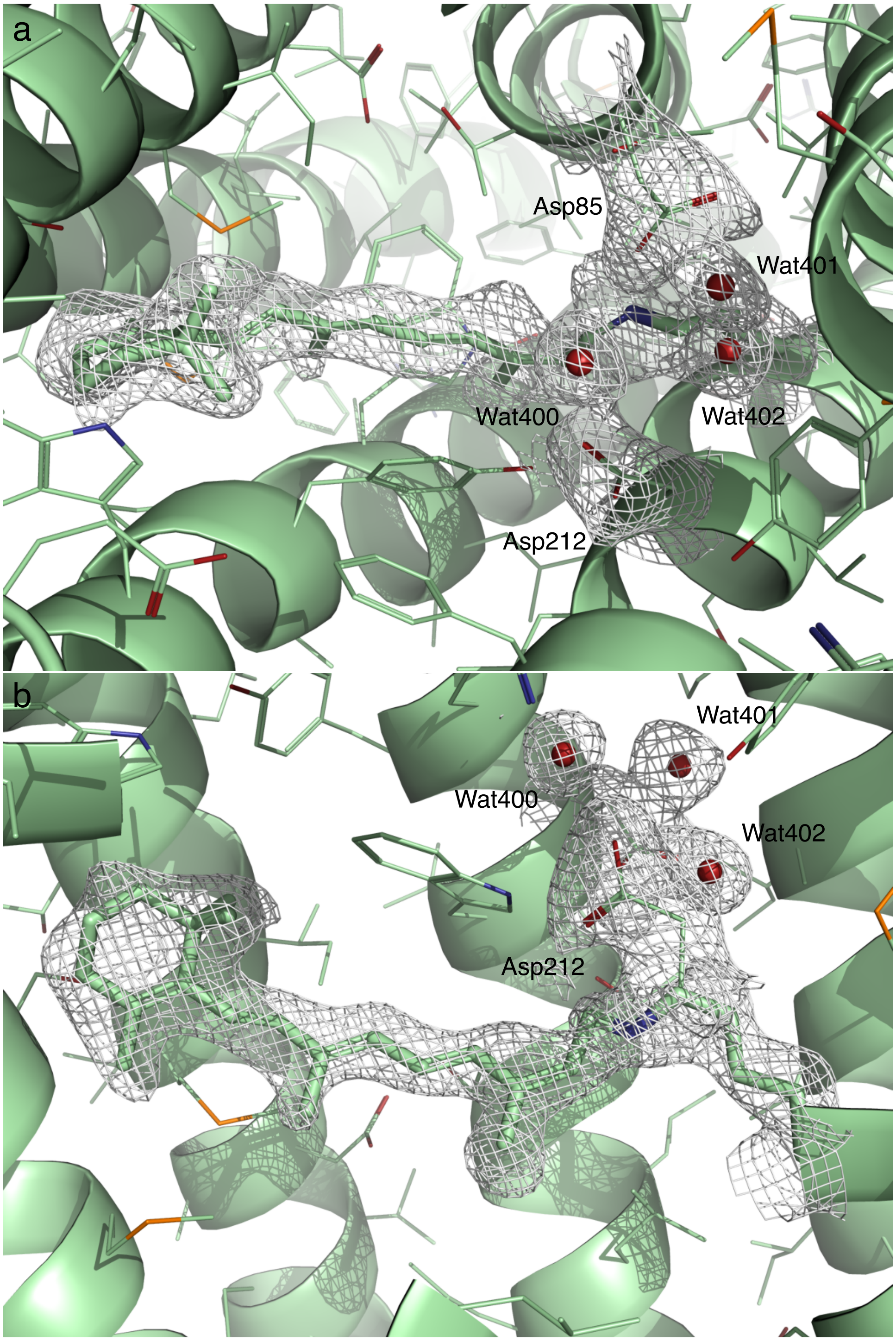

Figure S11. Two orthographical views of the 2Fo-Fc map of J′ contoured at 4σ. Here Fo is the reconstituted structure factor amplitudes rather than observed amplitudes (Table S2). Fc is the structure factor amplitudes calculated from the refine structure (Methods).

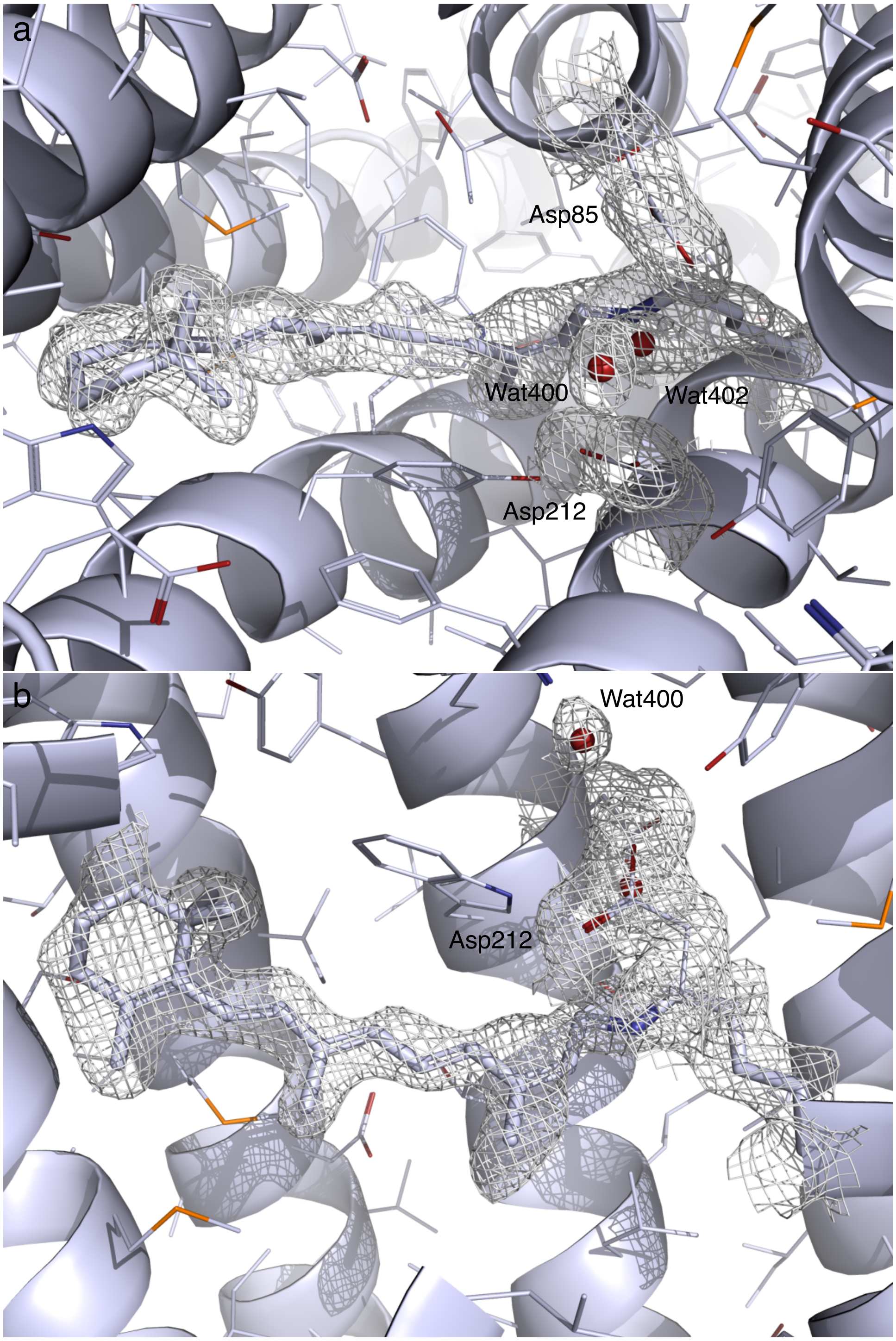

Figure S12. Two orthographical views of the 2Fo-Fc map of J contoured at 5σ. Here Fo is the reconstituted structure factor amplitudes rather than observed amplitudes (Table S2). Fc is the structure factor amplitudes calculated from the refine structure (Methods).
